# Mutualism between *Klebsiella* SGM 81 and *Dianthus caryophyllus* in modulating root plasticity and rhizospheric bacterial density

**DOI:** 10.1007/s11104-017-3440-5

**Published:** 2017-11-08

**Authors:** Shraddha Gang, Meenu Saraf, Christopher J. Waite, Martin Buck, Jörg Schumacher

**Affiliations:** 1 Department of Microbiology and Biotechnology, School of Sciences, Gujarat University, Ahmedabad, 380009 India; 2 Department of Life Science, Faculty of Natural Sciences, Imperial College, London, SW7 2AZ UK

**Keywords:** *Dianthus caryophyllus*, IAA, *Klebsiella*, Plant inoculation assays, Root plasticity

## Abstract

**Aims:**

*Dianthus caryophyllus* is a commercially important ornamental flower. Plant growth promoting rhizobacteria are increasingly applied as bio-fertilisers and bio-fortifiers. We studied the effect of a rhizospheric isolate *Klebsiella* SGM 81 strain to promote *D. caryophyllus* growth under sterile and non-sterile conditions, to colonise its root system endophytically and its impact on the cultivatable microbial community. We identified the auxin indole-3-acetic acid (IAA) production of *Klebsiella* SGM 81 as major bacterial trait most likely to enhance growth of *D. caryophyllus*.

**Methods:**

*ipdC* dependent IAA production of SGM 81 was quantified using LC-MS/MS and localised proximal to *D. caryophyllus* roots and correlated to root growth promotion and characteristic morphological changes. SGM 81 cells were localised on and within the plant root using 3D rendering confocal microscopy of *gfp* expressing SGM 81. Using Salkowski reagent IAA production was quantified and localised proximal to roots in situ. The effect of different bacterial titres on rhizosphere bacterial population was CFU enumerated on nutrient agar. The genome sequence of *Klebsiella* SGM 81 (accession number PRJEB21197) was determined to validate PGP traits and phylogenic relationships.

**Results:**

Inoculation of *D. caryophyllus* roots with *Klebsiella* SGM 81 drastically promoted plant growth when grown in agar and soil, concomitant with a burst in root hair formation, suggesting an increase in root auxin activity. We sequenced the *Klebsiella* SGM 81 genome, identified the presence of a canonical *ipdC* gene in *Klebsiella* SGM 81, confirmed bacterial production and secretion of IAA in batch culture using LC-MS/MS and localised plant dependent IAA production by SGM 81 proximal to roots. We found *Klebsiella* SGM 81 to be a rhizoplane and endophytic coloniser of *D. caryophyllus* roots in a dose dependent manner. We found no adverse effects of SGM 81 on the overall rhizospheric microbial population unless supplied to soil in very high titres.

**Conclusion:**

*Klebsiella* SGM 81 effectively improves root traits of *D. caryophyllus* in a dose dependent manner, likely through tryptophan dependent IAA production in the rhizoplane and potentially within the intercellular spaces of root tissue. Under optimal plant growth promoting conditions in non-sterile soil, the high total microbial titre in the rhizosphere supports a mutualistic relationship between *Klebsiella* SGM 81 and carnation that potentially extends to the wider rhizosphere microbiota.

**Electronic supplementary material:**

The online version of this article (10.1007/s11104-017-3440-5) contains supplementary material, which is available to authorized users.

## Introduction

Plant growth promoting rhizobacteria (PGPR) are a group of bacteria that are found in close vicinity of plant roots and enhance plant growth and development via a combination of various traits, including nitrogen fixation, phosphate solubilisation, plant hormone production, aminocyclopropane-1-carboxylate deaminase production and biolytic enzyme secretion (Bhattacharyya and Jha 2012; Bhardwaj et al. 2014; de Souza et al. 2015). PGPR can aggressively colonize the root structure and are often responsible for biocontrol against pests and pathogens in addition to plant growth promotion (Weller et al. 2002). Despite the ever increasing use of microbial formulations in order to improve plant growth yields, only a few studies have focused on the impact of microorganisms on ornamental plants, namely the application of *Azosprillium brasilense* (Zulfitri 2012) and *Cyanobacteria* (Shanan and Higazy 2009) on *Lavandula stoechas* and *Matthiola incana* respectively. Bacteria which colonise the regions in and around plant roots (rhizobacteria) are better able to modify, mobilize and/or solubilize nutrient supplements compared to other free-living organisms found in bulk soils (Hayat et al. 2010) microorganisms *Azosprillium brasilense* .

The production of the phytohormone indole acetic acid (IAA) by some PGPR is considered to be a direct mechanism by which microorganisms can modulate plant growth (Ali et al. 2009). Bacterial IAA can be produced via both tryptophan-dependant and tryptophan-independent pathways (Patten and Glick 1996). IAA exhibits both positive and negative effects on plant development (Mayak et al. 1999). In this way, although IAA plays an important role in enhancing plant root system development, it can inhibit root growth at extreme high or low concentrations. The effects of PGPR-produced IAA on root morphology and development, in particular with regards to specific IAA and inoculum concentrations, have been described previously (Dobbelaere et al. 1999). Rapid root development, inclusive of both elongation of primary roots and development of lateral and adventitious roots, increases the capacity of seedlings to adhere the soil and to acquire water and nutrients from their environment (Patten and Glick 2002a). In contrast to lateral and adventitious roots, which are induced by high IAA levels, primary root development is induced by generally low concentrations of IAA, typically in the nanomolar to picomolar range (Meuwly and Pilet 1991). It is thought that high IAA levels repress the primary root indirectly due to auxin-induced ethylene production (Peck and Kende 1995).

*Klebsiella* species exhibit plant growth promotion by various direct mechanisms, such as the ability to solubilise phosphate (Ahemad and Khan 2011), produce auxins (El-Khawas and Adachi 1999), fix atmospheric nitrogen (Mahl et al. 1965) and protect against abiotic stresses (Wu et al. 2014). *Dianthus caryophyllus*, commonly known as carnation, is one of the most economically important species in the *Caryophyllaceae* family, highly valued as an ornamental flower. It is available in a wide assortment of colours and patterns (Shiragur et al. 2004). In addition to its broad aesthetic appeal, the pharmacological potential of carnation has been extensively explored for anticancer, antiviral and other antimicrobial properties (Chandra et al. 2016). In this study, we show the beneficial effects of a *D. caryophyllus* rhizosphere *Klebsiella* isolate (SGM 81), identify the bacterial IAA anabolic pathway to promote *Dianthus*’ root growth in a dose dependant manner and link *Klebsiella* SGM 81 soil densities with plant phenotypes and the soil microbiome as a whole. No study has been reported for association of *D. caryophyllus* and *Klebsiella* to the best of our knowledge. We show *Klebsiella* SGM 81 to be an effective root coloniser of *D. caryophyllus,* both in the rhizoplane and the root interior, and discuss their specific mutualism within the wider rhizosphere ecology.

## Materials and methods

### Bacterial strain and culture conditions

*Klebsiella* SGM 81 was isolated from the rhizosphere of *D. caryophyllus* from Gujarat, India and was selected for the study considering the highest ability to produce IAA amongst all other isolates. The 16S rRNA sequence was deposited in gene bank under accession number KU748780. This isolate was grown in Nutrient broth (beef extract 1 g.L^−1^, yeast extract 2 g.L^−1^, peptone 5 g.L^−1^ and sodium chloride 5 g.L^−1^) for routine cultivation, and was preserved as glycerol stock at −80 °C for its maintenance. All the growth chemicals and growth media discussed in the study were procured from Sigma Aldrich, UK and VWR, UK.

### Isolation of *ipdC* gene by PCR

The *ipdC* gene (indole-3-pyruvate decarboxylase) from *Klebsiella* SGM 81 was amplified by polymerase chain reaction as described previously (Jha et al. 2012) with the following modifications. Specific primers were designed using *ipdC* gene data of *Klebsiella michiganensis* strain M5al (accession number AMPJ00000000) from NCBI with the following primer pair: Forward primer: 5′ TGATATCGCGTGGCGTTTGCCTGGTA3’ and reverse primer: 5′ GCGGATTTTCCCGGCGGTGTTCGTCG 3′ (Invitrogen, Thermofisher Scientific, UK). 25 μL reaction mixtures comprised: 2.5 μL of each primer (10 μM), 12.5 μL master mix (50 units/mL of Taq DNA polymerase, 400 μM dNTP, 3 mM MgCl2), 7.3 μL nuclease free water, and 0.2 μL of 10 ng genomic DNA (isolated using Zymo research genomic DNA isolation kit). PCR conditions were: initial denaturation at 95° for 5 min, followed by 35 cycles of 95° for 30 s, primer annealing at 60° for 30 s, extension at 72°for 2 min. This was followed by final extension at 72° for 10 min. Purity of the PCR product was assessed by electrophoresis on 0.8% agarose gel stained with SyBR safe DNA gel stain (Invitrogen, UK). PCR reactions were analysed by agarose gel electrophoresis and band of approximately 1.7 kb size (Supplementary Fig. 1), DNA gel extracted, sequenced (Genewiz, Essex, UK) and the deduced amino acid analysed (UniProt BLAST).

### IAA quantification and visualisation by Salkowski method

IAA production of *Klebsiella* SGM 81 was induced by supplementing Nutrient broth with 0.05%, 0.1%, or 0.15% (*w*/*v*) L-tryptophan and cultures were incubated in the dark on an orbital shaker at 200 rpm and 30 °C. IAA production and secretion was measured in culture supernatants at interval of 24 h till 96 h using Salkowski reagent as described previously described by (Jha and Saraf 2011). Briefly, 1 mL of culture supernatant was mixed with 1 mL of Salkowski reagent and incubated in the dark for 30 min. Development of pink colour was measured spectrophotometrically at 536 nm and IAA quantified using an IAA standard.

For in situ Salkowski staining of IAA in plant growth promoting assays, *D. caryophyllus* roots seven days after germination were dipped into SGM 81 suspensions of indicated titres, grown on agarose media for an additional two weeks and each root was stained with 400 μL Salkowski reagent for 30 min.

### Analysis of IAA by LC-MS/MS

IAA from culture supernatants was extracted as described (Jasim et al. 2013). Briefly, *Klebsiella* SGM 81 was inoculated into 200 mL of Nutrient broth supplemented with 0.5% of L-tryptophan and incubated for 4 days (bacterial density reaching approximately 8.0 log10 CFU.mL^−1^) at 30 °C at 200 rpm. Supernatants, following centrifugation at 5000 rpm for 20 min, were acidified to pH 2.5–3.0 with 1 N HCl before IAA was extracted with two volumes of ethyl acetate. IAA in the ethyl acetate phase was vacuum dried in a rotational evaporator at 40 °C and redissolved in 1 mL of methanol prior to storage at −20 °C. Identification of IAA was performed by subjecting the methanol extract to LC-MS/MS analysis on an Agilent 1100 LC system and an ABSciex 6500 Qtrap MS. Chromatography was on a Phenomenex Luna C18 column (100 mm × 2 mm x 3um). The identity of IAA in the samples was confirmed by Enhanced Product Ion scans. Data acquisition and analysis was performed with Analyst 1.6.1 software (AB Sciex).

### Plant experimental designs and conditions

Carnation seeds were obtained from an international seed suppliers Thompson & Morgan and were surface sterilised following the method of Bent (2006). Hundred seeds were allowed to germinate on Murashige and Skoog basal salt medium with 0.8% agarose, devoid of plant hormone supplements, until root lengths reached 2 cm. Twelve groups of five germinated plants were split into two sets. Both sets were subjected to each of the six treatments by immersing the root tips into 5 mL of the following for one hour: 10 μM bacterial IAA (T1), 10 μM synthetic standard IAA (T2), bacterial suspension with titres of 10^2^, 10^5^,10^8^ CFU mL^−1^ (T3 to T5 respectively), or sterile distilled water (TC). Treatments T1-T5 and TC will be used in all further description. The schematic presentation of experimental steps for plant study is also shown in Supplementary Fig. 2

### Plant growth experiments on agar

Germinated plants belonging to one set with all six treatments were transferred to MS agar medium devoid of IAA and sucrose in square petri plates (120 mm × 120 mm × 15 mm). The plates were kept at 25 °C in 14 h light and 10 h dark cycles in a plant culture room. Each plate carried 5 germinated seeds. Root architectures were observed after 21 days. Effect of *Klebsiella* SGM 81 was also observed on root length of model plant *Arabidopsis thaliana* col.-0 to validate its effect as root growth promoting agent.

### Soil experiments

For soil experiments, non-sterile soil was used. Compost soil (Levington F2 + Z seed and modular soil) was mixed with perlite (Levington soil and perlite) for good aeration in a 3:1 ratio. Soil/perlite mix was then transferred to pots to provide soil beds, and plants belonging to one set with each of six treatments were transferred to soil beds. Plants were uprooted after 21 days.

### Study of root architecture

Root architecture was studied in terms of primary root length, number of lateral roots, number of root hair/cm of primary root above the root apical meristem part of the root, fresh root weight and dry root weight and statistical comparisons were made as described by (Grossman and Rice 2012). The statistical analyses of the data was conducted using three replicates. Level of significance was studied using GraphPad prism 6 tool.

### Enumeration of root endophytic and rhizosphere bacterial population

Root endophytes were enumerated using method given by Tsavkelova et al. (2007) with slight modification where Roots were collected and subjected to three-step procedure: a 1 min wash in 70% ethanol, followed by a 1.5 min wash in 20% NaOCl, and a final rinse in sterile distilled water 3 times. This was followed by grounding them and serially diluting the crushed extract. The dilutions were then plated on nutrient agar plates and incubated overnight at 30 °C for colony formation. Rhizosphere bacteria were isolated on agar plates following method given by (Jha et al. 2010) with modification. Soil was collected from within the vicinity of 2 cm of root along with that adhered on root surface. This soil sample (1 g) was suspended in 9 mL of sterile normal saline and shaken at 200 rpm. Soil was allowed to settle, and the supernatant was serially diluted, plated on Nutrient agar plates and incubated overnight at 30 °C.

### GFP tagging and transformation

The plasmid pBBR1MCS4-*gfp* (5508 bp) was constructed for constitutive expression of green fluorescence protein (*GFP*) in *Klebsiella* SGM 81. Briefly, the *gfp* (mut3b) gene (Cormack et al. 1996), preceded by a synthetic sigma-70 promoter (BBa_J23104) and ribosome binding site (BBa_B0030) sourced from the Registry of Standard Biological Parts (http://parts.igem.org), was cloned into the broad host range vector pBBR1MCS4 (Kovach et al. 1994) as an SphI-SacI restriction fragment. pBBR1MCS4-*gfp* was transformed by electroporation into *Klebsiella* SGM 81 for fluorescence visualisation in plant tissue. We found that *Klebsiella* SGM 81 did not readily transform using standard protocols for *Escherichia coli*, presumably due to high expression of exo-polysaccharides. Therefore, overnight cultures of *Klebsiella* SGM 81 were re-inoculated in LB broth containing 0.7 mM EDTA and cells harvested when an OD_600_ of 0.5 was reached. The transformation was then conducted as described previously (Fournet-Fayard et al. 1995). Gene knockout experiments to create mutant of *ipdC* gene was also done by targeted mutagenesis using red recombinase. Bacterial strain was subjected to transformation using red expressing plasmid pKD46 using method given by (Datsenko and Wanner 2000). However attempt to create knockout failed presumably due to low transformation frequency of *Klebsiella* SGM 81.

### Root colonisation microscopy

Root colonisation by *GFP*-tagged bacteria was studied after 7 days of inoculation, when roots were collected and surface sterilised following method given by (Tsavkelova et al. 2007) with slight modifications as discussed above in enumeration of bacteria section. Roots were subjected to three-step procedure: a 1 min wash in 70% ethanol, followed by a 1.5 min wash in 20% NaOCl, and a final rinse in sterile distilled water 3 times. Roots were cut into sections of 1 cm and dipped in 10 mg.mL^−1^ of propidium iodide to stain the plant root cells. Bacterial colonisation images were acquired with a Zeiss LSM 510 confocal microscope, using a 63× Plan-Apochromat 1.40 objective, with a z-step of 0.7 μm and a 70 nm pixel size. Micrographs were prepared with Icy (de Chaumont et al. 2013).

### Identification of *Klebsiella* SGM 81

Genomic DNA isolation and 16 s rRNA sequencing of *Klebsiella* SGM 81 was carried out by Chromous Biotech, Banglore, India. The sequence obtained was submitted to NCBI gene bank and accession number was received. Genome sequencing of *Klebsiella* SGM 81 was carried out by Microbes NG, Birmingham, UK. The downstream processing of sequenced data was accomplished using several bioinformatics tools. Briefly Genome assembly was carried out using the BugBuilder pipeline (http://www.imperial.ac.uk/bioinformatics-data-sciencegroup/resources/software/bugbuilder/). Contig sequences were scaffolded against the reference genome sequence of *Klebsiella oxytoca* strain CAV1374 (CP011636.1) using the mauve scaffolder (Darling et al. 2010), followed by gap closure using Pilon (Walker et al. 2014). The assembled genomes were then annotated using Prokka (Seemann 2014) The presence or absence of genes was determined by carrying out blastp searches (e = 0.01) (Camacho et al. 2009) against the predicted proteins from the annotated genome, using homologous genes from *Klebsiella* SGM 81 sequences. To produce phylogenetic trees, available high quality 16 s RNA sequences were obtained from the RDP database (Cole et al. 2005), and a multiple alignment produced using muscle (Edgar 2004). Ambiguous regions were removed from the alignment with BMGE (Criscuolo and Gribaldo 2010) and a bootstrapped maximum likelihood tree constructed using RaXML (Stamatakis 2014), with a GTRCAT model and 10,000 bootstrap iterations.

## Results

### Tryptophan dependent pathway of IAA production of by *Klebsiella* SGM 81

*Klebsiella* SGM 81 was isolated from the *D. caryophyllus* rhizosphere during a screen for PGPR. A 16S rRNA gene sequence analysis indicated that the isolated strain belongs to the *Klebsiella* genus, and was named SGM 81*.* We observed the presence of *Klebsiella* SGM 81 stimulated lateral root hair formation of *D. caryophyllus* seedlings, in a manner dependent on tryptophan in the growth media (see below). The *ipdC* gene, coding for indole-3-pyruvate decarboxylase, is generally associated with converting tryptophan into indole-3-acetic acid (IAA) (Persello-Cartieaux et al. 2003), a product which is linked to the stimulation of lateral root growth in a variety of plants (Gravel et al. 2007). We amplified a 1.7 Kb DNA fragment from genomic *Klebsiella* SGM 81 DNA using primers specific to the *ipdC* gene in the model *Klebsiella michiganensis* strain M5al genome sequence (AMPJ00000000). The identity of the amplified fragment to code for *ipdC* in *Klebsiella* SGM 81 was confirmed by DNA sequencing, with the deduced amino acid sequence showing 99.8% similarity with the predicted protein sequence of *Klebsiella* sp. NFIX56 (NCBI 1566183) (Supplementary Fig. 3).

**Fig. 1:**
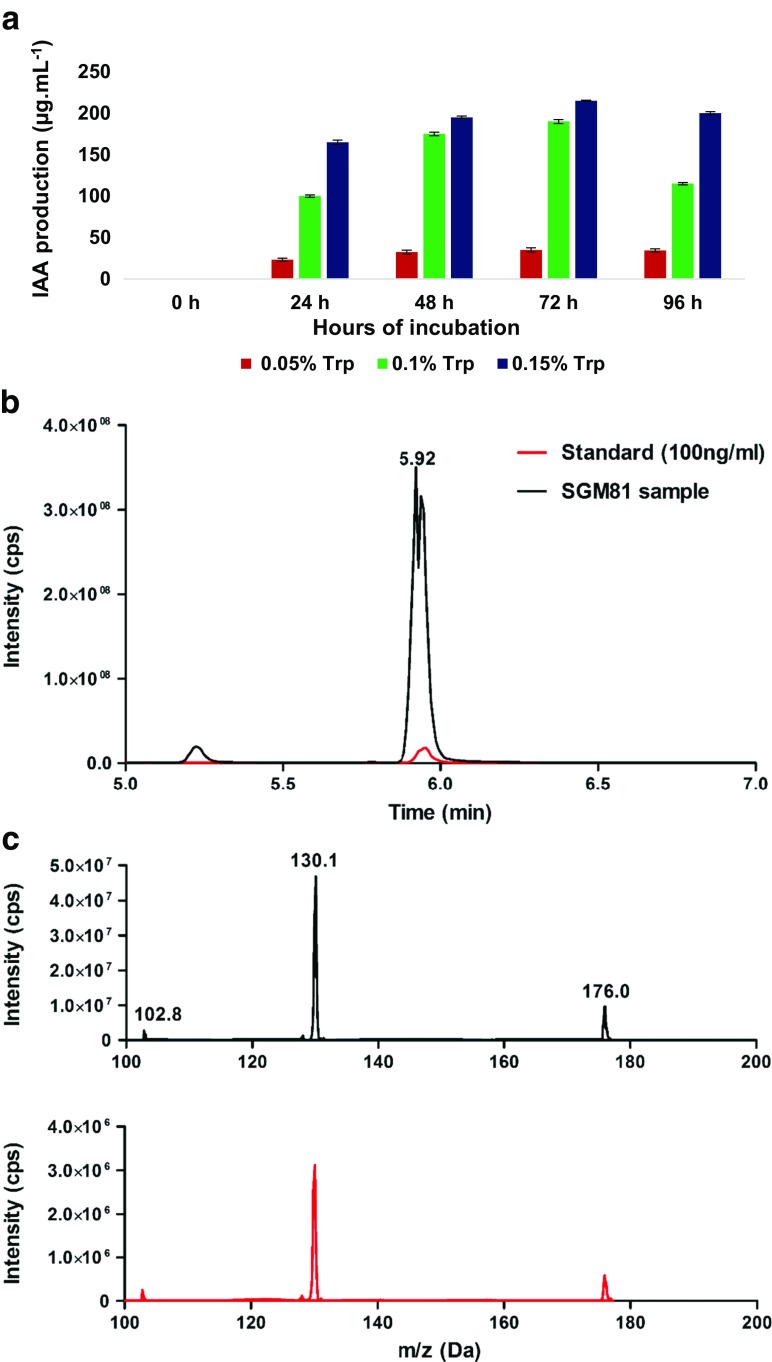
IAA detection and quantification using Salkowski reagent and LC-MS/MS. **a** shows mean values of three replicates for quantitative estimation of IAA by Salkowski reagent after 0 h (no inoculation) and 24–96 h (after inoculation). Error bar represent one SD (*n* = 3). **b**, **c** show LC-MS/MS plot of commercial standard control and SGM 81 supernatant sample, showing retention times in upper panel (**b**) and m/z ratios in lower panel (**c**)

Next, we assessed the tryptophan dependent production and secretion of IAA from *Klebsiella* SGM 81. Batch cultures of *Klebsiella* SGM 81 were grown in the presence and absence of tryptophan and cell supernatants analysed for IAA using both the methods of Salkowski (Jha and Saraf 2011) and LC-MS/MS (Fig. 1). Using the former we found apparent IAA production increased with increasing tryptophan concentrations (Fig. 1a). No IAA production was found prior to inoculation (0 h) indicating that *Klebsiella* SGM 81 cells are responsible for this reaction. We found a maximum yield of IAA after 72 h with 0.15% tryptophan (215 μg.mL^−1^), but relatively high yields after 24 h suggest a high rate of IAA production during the first day. Tryptophan alone had no negative effects on *Klebsiella* SGM 81 growth, in fact a slight positive growth effect, excluding the possibility that growth differentials could account for different IAA production rates (Supplementary Fig. 4). IAA yields decreased after 96 h, potentially due to stationary phase cells and/or potential nutrient exhaustion.

To confirm the molecular identity and assess the purity of IAA produced and secreted by *Klebsiella* SGM 81, IAA was extracted from cell supernatant and subjected to LC-MS/MS. Overlapping retention times during LC (5.90 min and 5.92 min) and identical m/z ratios (130.1) between a commercial IAA standard and sample supernatants showed *Klebsiella* SGM 81 to produce substantial amounts (960 μg*mL^−1^ after 72 h) of relatively homogeneous IAA when grown in the presence of 0.5% tryptophan (Fig. 1b). Since we could not detect IAA in the absence of tryptophan, we conclude that *ipdC* is highly substrate specific and that intracellular tryptophan concentrations of *Klebsiella* SGM 81 are insufficient to produce detectable amounts of IAA. These results may suggest that IAA is most readily produced in the vicinity of plant roots where tryptophan is likely to be present from root exudates, rather than in the bulk soil (Karnwal 2009).

### Root growth promotion by *Klebsiella* SGM 81

**Fig. 2:**
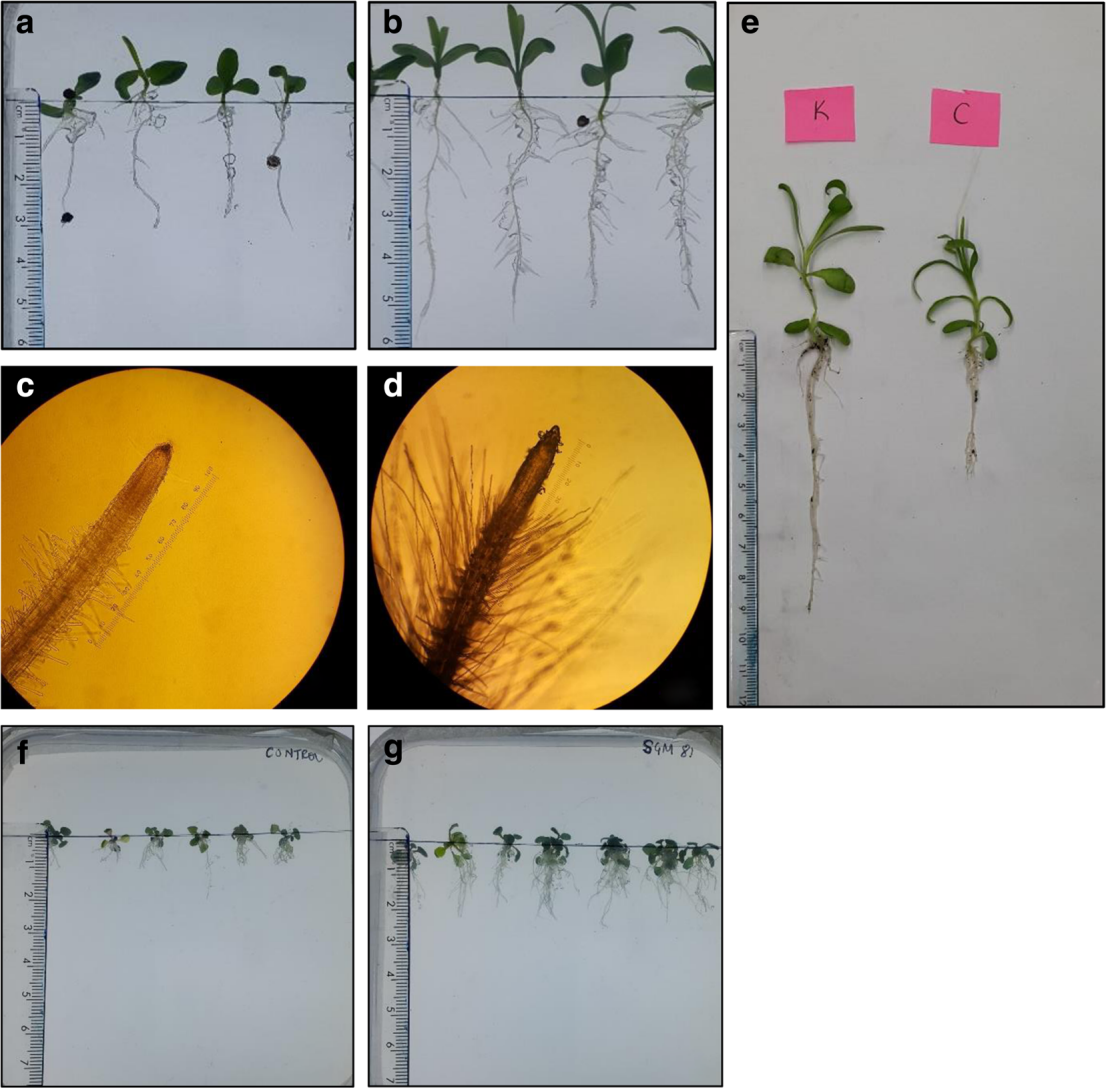
Root morphology of *D. caryophyllus* inoculated with *Klebsiella* SGM 81. Phenotypic difference in root length and number of root hairs in non-inoculated control and treated plants after 21 days. Image shows control plant (**a**), root length when treated with 10^2^ CFU.mL^−1^ Klebsiella (**b**). Light microscopy images of root hairs in non-inoculated control (**c**), and root hairs when treated with 10^2^ CFU.mL^−1^ Klebsiella SGM 81 (**d**). Root lengths of non-inoculated control (left) and inoculated (10^5^ CFU.mL^−1^) *D. caryophyllus* (right) grown in soil (**e**). Root length of non-inoculated control and treated plants of *Arabidopsis thaliana* col.-0 (**f**, **g**)

Originally selected isolated for plant growth promoting properties, we investigated *Klebsiella* SGM 81 plant growth promotion of *D. caryophyllus* in more detail, both in vitro (agarose plates) and in soil with 6 different treatments: 10 μM SGM 81 produced IAA (T1), 10 μM commercial standard IAA (T2), SGM 81 suspension with titres of 10^2^, 10^5^,10^8^ CFU mL^−1^ (T3 to T5 respectively), and sterile distilled water (TC). To validate the effect of IAA on *D. caryophyllus* we supplemented agarose media with a final synthetic IAA (10 μM) or the same *Klebsiella* SGM 81 supernatants used for LC-MS/MS (for final 10 μM IAA). Both treatments induced significant and highly similar phenotypic alterations in root architecture when compared to the water control (Table 1), particularly with respect to increased lateral root development, root hair formation and root fresh weight, with also modest but significant increases in root length and root dry weight. Inoculation of plant roots with different titres of bacterial cells induced similar, but more pronounced root morphological changes (Fig. 2 and Table 1). These results suggest that externally applied IAA causes important root morphological changes in *D. caryophyllus* and imply that IAA production of *Klebsiella* SGM 81 could largely account for its plant growth promotion properties of *D. caryophyllus.* Differences in root morphological traits were observed when comparing both different inoculum titres and different environments (agarose and soil). The most striking plant phenotypic outcome was the drastically enhanced number of root hairs in plants treated with low (T3) and medium (T4) bacterial cell titres. The T3 treatment has the strongest effect in agarose while the T4 had the strongest impact in soil. Similar results were observed with *Arabidopsis thaliana* (Fig. 2f, g). The roots of Arabidopsis plants treated with *Klebsiella* SGM 81 were longer and had more lateral roots compared to non-treated roots. However, inoculation of *D. caryophyllus* with a high titre (T5) of *Klebsiella* SGM 81 had a very strong negative effect on plant root and plant development in both experimental environments (below).

**Table 1:** Measurements of root phenotypes in differently treated plants

Root architecture	Treatments					Control
	T1 Synthetic IAA	T2 Bacterial IAA	T3 10^2^ CFU.mL^−1^	T4 10^5^ CFU.mL^−1^	T5 10^8^ CFU.mL^−1^	TC Distilled water
Primary root length(cm)						
MS agar Soil *p* value	4.90 ± 0.17^b^ 4.90 ± 0.00^d^ ns	4.97 ± 0.29^b^ 5.07 ± 0.15^c^ ns	5.03 ± 0.15^c^ 5.97 ± 0.21^d^ <0.0001	4.40 ± 0.17^a^ 9.07 ± 0.06^d^ <0.0001	1.83 ± 0.06^d^ 2.03 ± 0.06^d^ ns	3.97 ± 0.12 4.03 ± 0.06 ns
No. of lateral roots						
MS agar Soil *p* value	20.33 ± 0.58^d^ 18.63 ± 1.53^e^ ns	23.67 ± 0.58^d^ 20.67 ± 0.58^b^ <0.05	34.33 ± 1.53^d^ 21.00 ± 1.00^b^ <0.0001	18.33 ± 0.58^d^ 51.00 ± 2.65^d^ <0.0001	4.67 ± 1.00^c^ 8.00 ± 1.00^b^ <0.05	10.67 ± 0.58 15.33 ± 1.53 <0.001
No. of root hairs						
MS agar Soil *p* value	30.67 ± 0.58^d^ 31.33 ± 1.53^d^ ns	30.67 ± 0.58^d^ 32 ± 1.00^d^ ns	85.67 ± 0.58^d^ 74.33 ± 0.58^d^ ns	74.67 ± 0.58^d^ 117.0 ± 0.58^d^ <0.0001	0.67 ± 0.58^c^ 5.00 ± 0.00^d^ ns	8.33 ± 1.15 15.33 ± 0.58 ns
Fresh root weight(g)						
MS agar Soil *p* value	0.47 ± 0.01^c^ 1.03 ± 0.06^c^ <0.0001	0.47 ± 0.01^c^ 0.93 ± 0.03^d^ <0.0001	0.43 ± 0.02^b^ 1.17 ± 0.03^d^ <0.0001	0.51 ± 0.03^c^ 1.49 ± 0.01^d^ <0.0001	0.1 ± 0.00^c^ 0.81 ± 0.01^d^ <0.0001	0.27 ± 0.03 0.54 ± 0.00 <0.0001
Dry root weight(g)						
MS agar Soil *p* value	0.16 ± 0.002^e^ 0.36 ± 0.00^d^ <0.0001	0.16 ± 0.004^b^ 0.31 ± 0.012^d^ <0.0001	0.21 ± 0.01^c^ 0.58 ± 0.025^d^ <0.0001	0.15 ± 0.00^e^ 0.76 ± 0.015^d^ <0.0001	0.05 ± 0.00^c^ 0.41 ± 0.01^d^ <0.0001	0.14 ± 0.013 0.17 ± 0.006 <0.05

Effect of five treatments (Synthetic IAA, bacterial IAA, 10^2^, 10^5^, 10^8^ CFU.mL^−1^) on root architecture. Statistical analysis was done using GraphPad Prism 6 software calculated at *p* ≤ 0.05. Data represents mean values of three replicates with standard deviation. Significance of data has been analysed for significant difference in terms of: (i) treatment vs control mentioned through alphabets a (≤ 0.05), b (≤ 0.01), c (≤0.001) d (≤0.0001) and e (not significant). (ii) MS agar vs Soil mentioned through *p* values

### *Klebsiella* SGM 81 produces IAA proximal to plant root

**Fig. 3:**
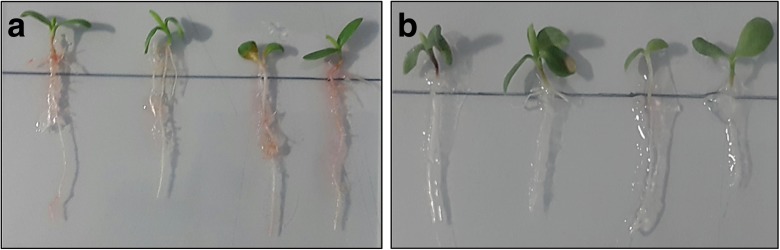
In situ Salkowski staining on plant roots treated with different *Klebsiella* SGM 81 and control plants. Visual localisation of IAA using Salkowski reagent on roots of *Dianthus caryophyllus* treated with: **a** 10^2^ CFU.mL^−1^, **b** Control plants. The development of pinkish red colour proximal to root indicates the presence of auxin. The control plants do not show pink colour upon addition of the reagent which indicates the absence of any exogenous bacterial IAA

Next we showed that IAA is produced in the SGM 81-*Dianthus* plant growth promotion experiments using Salkowski reagent on plants. We stained grown plants with 400 μL of Salkowski reagent to observe any visible colour change indicating presence of IAA. Figure 3 and Supplementary Fig. 5 show the development of a pinkish colour proximal to roots, indicating the presence of auxin. Maximum colour development was observed in 10^2^ CFU.mL^−1^ (Fig. 3a) and 10^5^ CFU.mL^−1^ inoculated plants (Supplementary Fig. 5a), correlating with best plant root phenotypes (Table 1). The plant roots treated with 10^8^ CFU.mL^−1^ (Supplementary Fig. 5b) showed adverse plant growth effects and no significant pink colour development. The non-inoculated control plants also did not show pink colour, indicating that the source of the IAA is associated with bacterial colonisation (Fig. 3b). We confirmed on plant free agarose inoculated plates that IAA production by SGM 81 was strictly tryptophan dependent, as it was for batch cultures (Supplementary Fig. 3c,d).

### Endophytic colonization of cultivable bacteria in *D. caryophyllus*

**Fig. 4:**
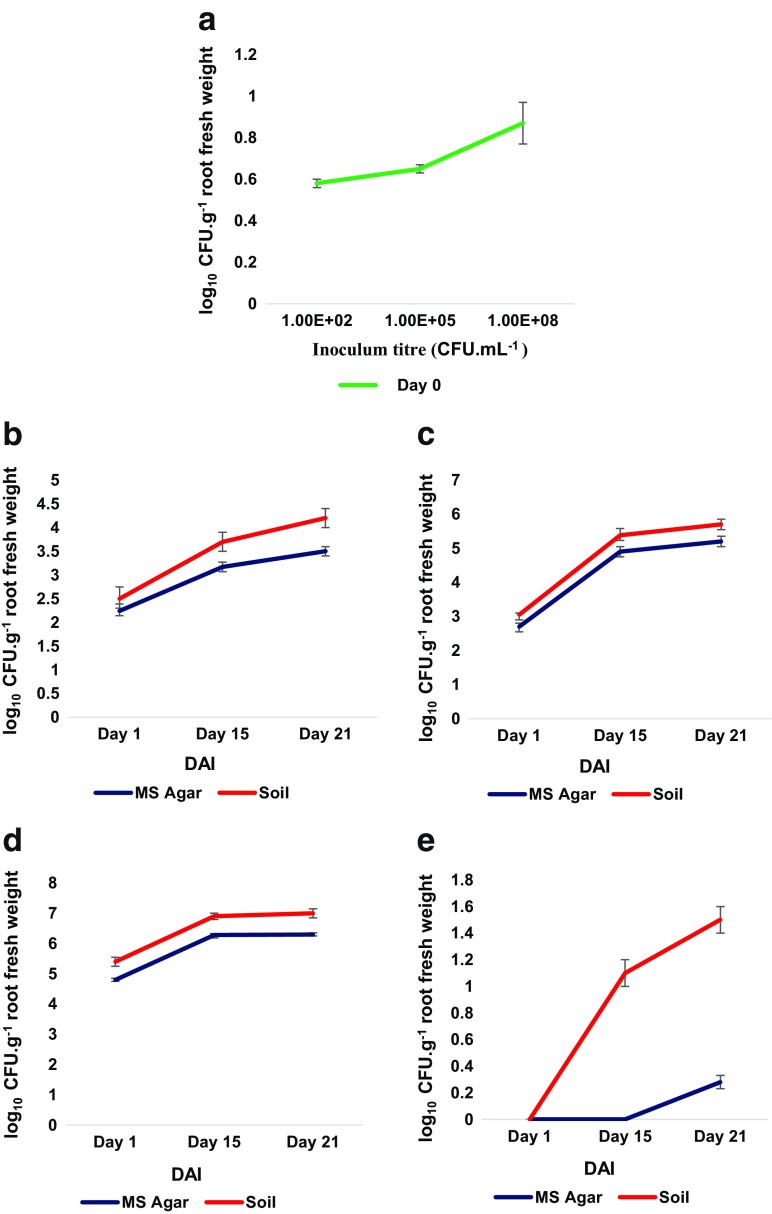
Enumeration of endophytic bacteria. The endophytic growth of cultivable bacteria in *D. caryophyllus* by isolating on Day 0 (**a**) and at DAI 1, 15, 21 from plants treated with 10^2^ CFU.mL^−1^(**b**), 10^5^ CFU.mL^−1^ (**c**), 10^8^ CFU.mL^−1^ (**d**) and control plants (**e**). Error bars represent one standard deviation from the mean (n = 3)

**Fig. 5:**
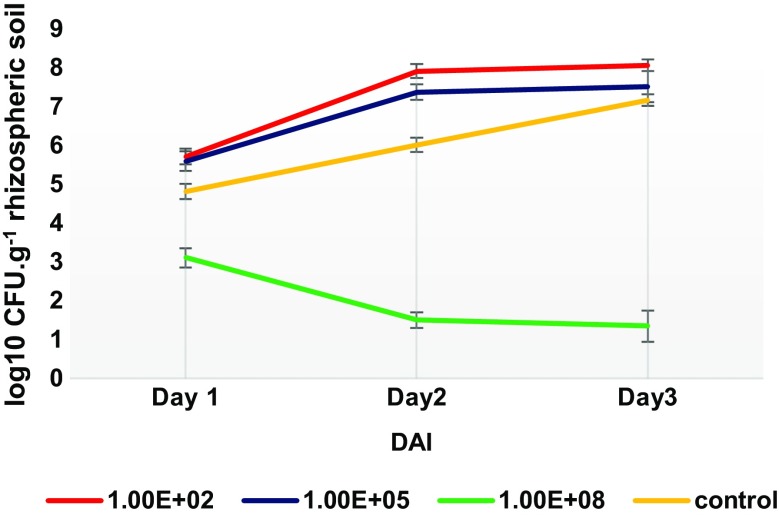
Enumeration of rhizosphere bacteria. Rhizosphere bacteria densities were calculated by isolating bacteria after DAI 1, 15, 21 from soil around and adhered to plant roots treated with 10^2^, 10^5^, 10^8^ CFU.mL^−1^ and control (untreated) plants . Error bars represent one SD from the mean (n = 3). *P* value ≤0.05 for 10^2^, 10^8^ CFU.mL^−1^ vs control and ≥0.05 for 10^5^ CFU.mL^−1^ vs control

The microbial density and diversity in the rhizosphere is thought to substantially contribute to plant growth and health. The bacterial titre dependent positive and negative impact of *Klebsiella* SGM 81 to the growth of *D. caryophyllus* is likely due to its impact on the rhizospheric ecology, comprising microbial composition in non-sterile conditions (soil) and/or plant responses to the addition of *Klebsiella* SGM 81. We show that *Klebsiella* SGM 81 colonises *D. caryophyllus* root tissue (below) and can proliferate endophitically. To evaluate the impact of *Klebsiella* SGM 81 on the *D. caryophyllus* rhizosphere ecology*,* we enumerated over time the densities of bulk soil and endophytic bacterial populations following inoculation with *Klebsiella* SGM 81. After uprooting and sterilising plants, root extracted cultivable bacteria were counted using colony forming units (CFU) and counts normalised against root fresh weight (Fig. 4). Rhizospheric cultivable bacteria were similarly enumerated per gram weight of soil (Fig. 5). While such methods are semi quantitative at best and do not, for example, enumerate non-cultivatable microbes, we obtained sufficiently consistent enumeration outcomes that may speak to the plant growth outcomes.

The much higher counts of endophytic bacteria after *Klebsiella* SGM 81 inoculation in sterile MS agar medium (Fig. 4 b-d) compared to water only control (Fig. 4e) suggested that under these conditions *Klebsiella* SGM 81 sustained the endophytic lifecycle in *D. caryophyllus* following the inoculation. We attribute the presence of some endophytic bacteria in the water control at 21 DAI either through external contamination or presence of bacteria within the seed (Mastretta et al. 2009). Under all conditions, endophytic populations increased over time, indicating that there is no obvious effective plant response to limit endophytic growth. Endophyte numbers increased with increasing titres, but not linearly. The difference in the population isolated on day 1 from T3 and T4 vs T5 plants is unclear, however, the high endophyte concentration following T5 treatment may be a consequence of damage in root structure due to high bacterial titre, as seen in studies with bacteria *Pseudomonas fluorescens* SS101 and *Paenibacillus polymyxa* (Timmusk et al. 2005). In summary, inoculation with increasing *Klebsiella* SGM 81 titres increases endophytic and soil colonisation, but with highest numbers of endophytes seen under conditions that are deleterious to plant root development (T5), while lower colonisation levels correlated with promoted root growth. No obvious or effective plant defence to limit high endophyte populations was observed although it remains to be shown if the endophytic lifestyle accounts for both beneficial and/or adverse plant growth effects.

### Effect of *Klebsiella* SGM 81 on population of rhizosphere bacteria

We enumerated CFU in the rhizosphere by isolating the soil attached to and around root surface after uprooting. As shown in (Fig. 5), highest bacterial density was extracted from the rhizosphere of plants subject to the T3 treatment (log_10_ 8.05 CFU.g^−1^ soil) and lowest in case of T5 treatment (log_10_ 1.34 CFU.g^−1^ soil). Strikingly, the rhizosphere of untreated plants had comparatively a denser population than T5 treatment in soil (Fig. 5), which gradually decreased over several orders of magnitude. In contrast, the treatments T3 and T4, conditions which correlated positively with plant root growth promotion, the rhizospheric population grew steadily over time. These results clearly suggest that appropriate dose of *Klebsiella* SGM 81 plays important role in maintaining and increasing rhizosphere bacteria population and it may inhibit mutualistic plant-rhizobacteria communities at higher titre. In control plants, the bacterial density increases gradually till 21 days, indicating that in absence of external inoculant a growing population of bacteria can be supported in the rhizosphere region as the plant ages. Overall, these findings strongly suggest that *D. caryophyllus* tolerates and perhaps nurtures bacterial populations in the rhizosphere but not when subject to very high titres of *Klebsiella* SGM 81. We can currently not distinguish whether the decrease of rhizosphere microbial titres under treatment T5 is a consequence of poor plant health, in which case fewer nutrients such as sugars might be exuded into the soil, and/or whether high microbial titres may elicit plant defence mechanisms (e.g. MAMPS) that could involve the secretion of antimicrobial metabolites, which were shown to be excreted from *Ocimum basilicum* roots upon challenge from the pathogen *Pythium ultimum* (Bais et al. 2002). The observation that endophytic bacterial titres did not decline when *D. caryophyllus* when exposed to high *Klebsiella* SGM 81 titres but the rhizosphere titres did decline may provide some evidence that a lack of nutrient supply from a healthy plant, rather than an active plant defence accounts for the rhizosphere bacterial decline.

### *Klebsiella* SGM 81 adheres the root epidermis and pericycle apoplast regions

**Fig. 6:**
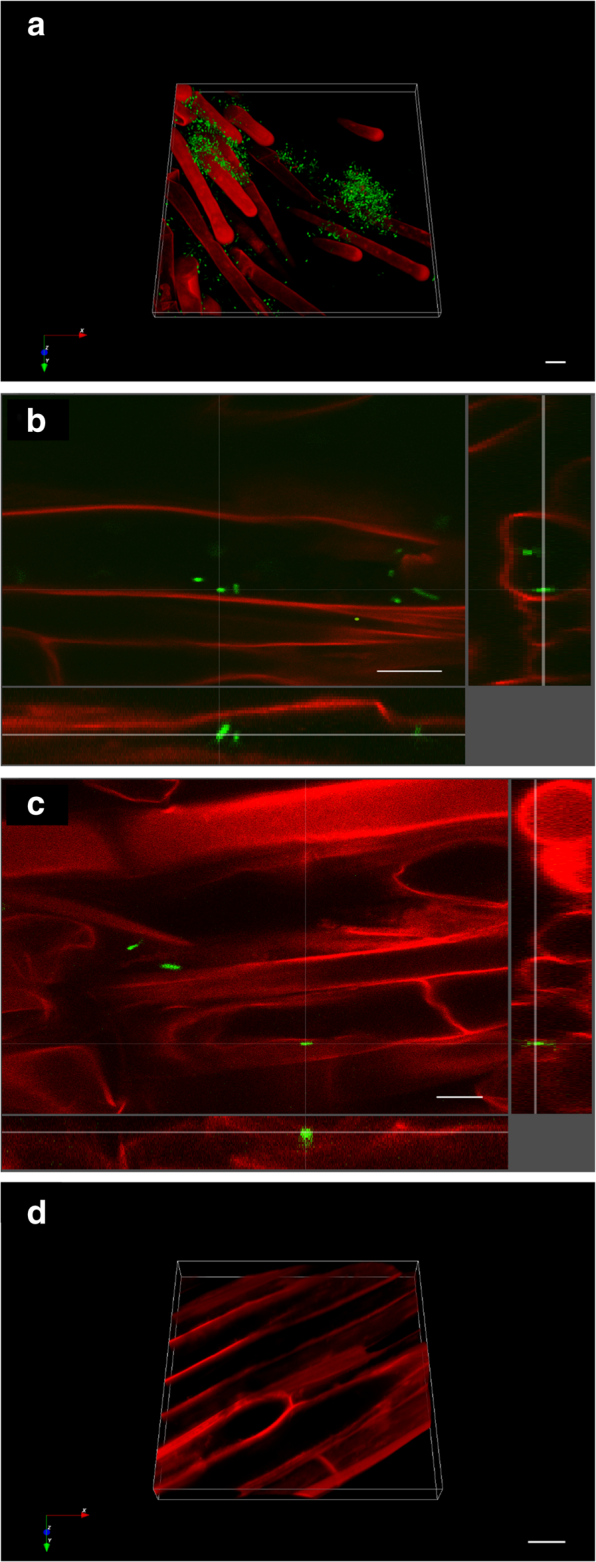
Microscopy of root tissue to localise *gfp* tagged *Klebsiella* SGM 81. Colonisation of Carnation roots by *gfp* tagged *Klebsiella* SGM 81. Confocal microscopy was performed using 1 cm long root section. Images showing 3D rhizoplane (a), within epidermal *D. caryophyllus* cells (b), the root apoplasm (c) and non-treated control root cells and rhizoplane (d). Cross hair analysis of 3D images (Fig. 6b and c) indicate that SGM 81 localises rhizoplane and apoplast respectively. Scale bar: 10 μm

Enumeration of viable bacteria of plants grown in MS-agar and non-sterile soil, suggested both epiphytic and endophytic lifestyles of *Klebsiella* SGM 81 with *D. caryophyllus*. To confirm an endophytic lifestyle and better evaluate the beneficial traits of *Klebsiella* SGM 81*,* we visualised their presence on and within *D. caryophyllus* roots, using confocal microscopy (Fig. 6). We constructed a broad host range based vector pBBRMCS4-*gfp*, constitutively expressing *gfp*, which was transformed by electroporation into *Klebsiella* SGM 81 for visualisation of live cells. Following root inoculation with the intermediate titre of *Klebsiella* SGM 81 and 7 days growth on MS-agar, roots were stained with propidium iodide. Bright field, red and green florescence images were taken to visualise roots, cell walls and *Gfp*-tagged bacteria, respectively. We found *Klebsiella* SGM 81 to be localised extensively at the rhizoplane (Fig. 6a), and also within epidermal *D. caryophyllus* cells (Fig. 6b, Supplementary Fig. 6a) and the root apoplast (Fig. 6c, Supplementary Fig. 6b). We did not detect any green fluorescent bacteria in or on non-inoculated roots (Fig. 6d, Supplementary Fig. 6c). We conclude that *Klebsiella* SGM 81 is an excellent rhizoplane coloniser and also endophyte of *D. caryophyllus*.

### *Klebsiella* SGM 81 genome sequencing and phylogeny

**Fig. 7:**
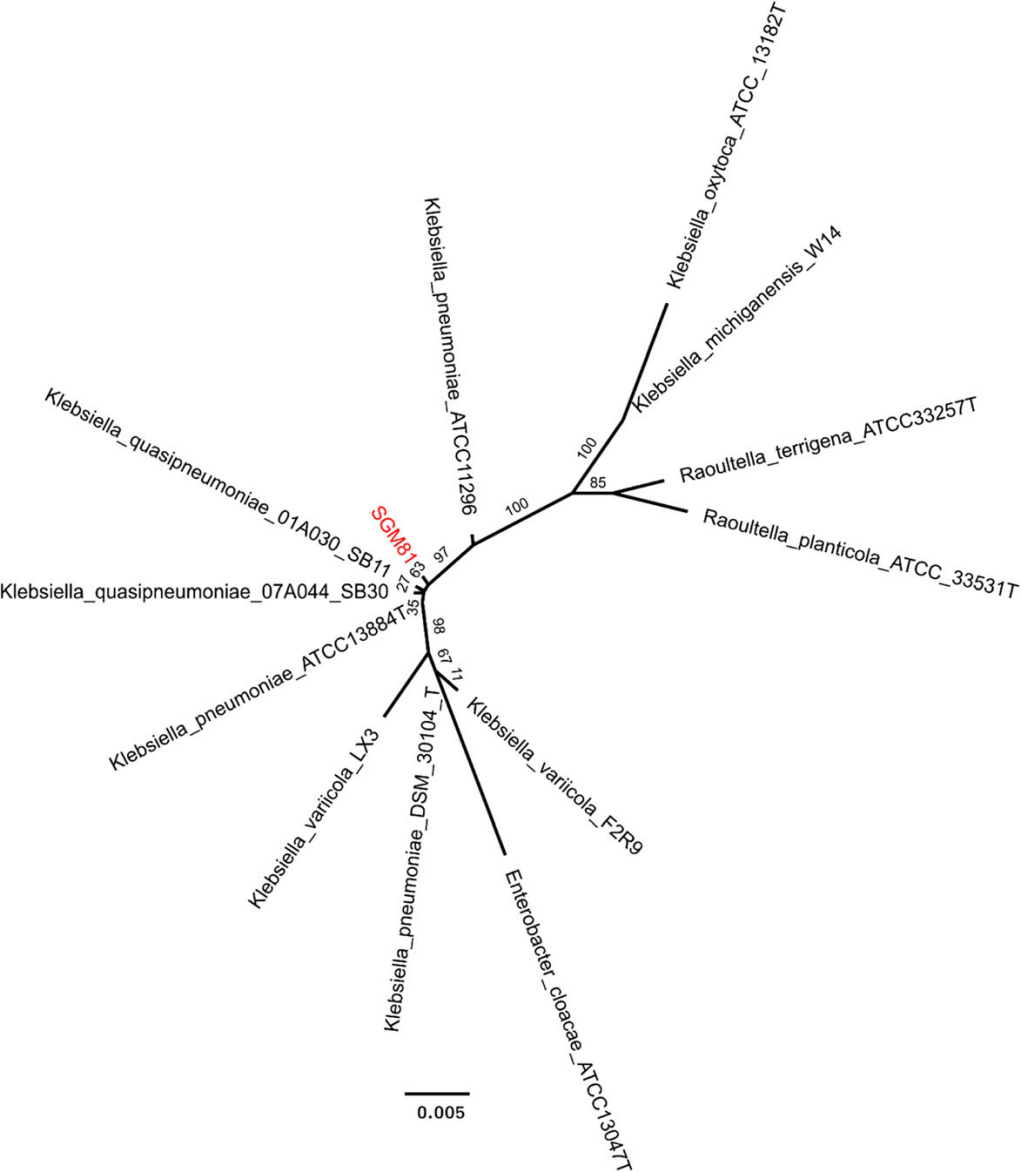
Phylogenetic tree of *Klebsiella* SGM 81 based on aligned 16 s rRNA sequences. The subject strain is highlighted in red colour. The tree was produced using 10,000 bootstrap iterations. The numbers are the bootstra*p* values giving an idea of the confidence in the placement of the taxa. The nearest member strain of *Klebsiella* SGM 81 is *Klebsiella quasipneumonae*

We carried out a 16S RNA phylogenetic analysis and phylogenetic tree was produced based on the 16S sequence of accession KU748780 at various scales. It was identified as *Klebsiella quasipneumonae* based on sequence homology (Fig. 7). The percent homology of *Klebsiella* SGM 81 to other nearest *Klebsiella quasipneumoniae*_01A030 is 94% and that with *Klebsiella quasipneumoniae*_07A044 is 93.7%. We determined the whole genome sequence of *Klebsiella* SGM 81 and complete genome sequence have been submitted (accession number PRJEB21197) to the European Bioinformatics Institute (EMBL-EBI). Details are provided in the “Materials and methods” section and supplementary table. We inspected the *Klebsiella* SGM 81 genome for the presence of *rmpA* and *rmpA2* genes, coding for capsule formation and indicative for *Klebsiella pneumonia* type 1 strains that are most frequently associated with human infections. The absence of these genes in *Klebsiella* SGM 81 genome suggests limited pathogenicity potential (Holt et al. 2015).

## Discussion

Bacteria of the genus *Klebsiella* have been found to associate with roots of several agriculturally important crops, in some cases endophytically, and have been shown to promote plant growth via auxin production (El-Khawas and Adachi 1999) and nitrogen fixation (Gyaneshwar et al. 2001). Here we show that *Klebsiella* SGM 81, endogenous to the rhizosphere of *D. caryophyllus*, strongly promotes its host’s root development in the first weeks of plant growth. The primary root length (cm) of plants treated with synthetic IAA and bacterial IAA is nearly 23% longer than the primary root length of untreated plants. Plants treated with 10^5^ CFU.mL^−1^ of bacteria grown in soil yielded 125%, 232%, 663%, 347% greater outcomes than control plants for primary root length, number of lateral roots, number of root hairs and dry root weight, respectively. Several fold increases in plant root attributes when inoculated with plant growth promoting rhizobacteria have been reported, for example for *Streptomyces* on rice (Harikrishnan et al. 2014) and *Kocuria turfanensis* on groundnut (Goswami et al. 2014). It was previously unclear if these root morphological changes are a general or a specific feature of beneficial *Klebsiella*-plant systems, however, our similar findings with both *D. caryophyllus* and *A. thaliana* may suggest the former. Plant growth promoting phenotypes with both endogenous and exogenous *Klebsiella* species have been reported, for instance, the enhancement of wheat growth during abiotic stress (Singh et al. 2015), the capacity for nitrogen fixation in wheat (Riggs et al. 2002) and the enhancement growth of *Salicornia bigelovii* by (Rueda-Puente et al. 2003). However, it remains generally challenging to attribute specific bacterial plant growth promoting properties or combinations thereof e.g. phosphate solubilisation, IAA production, nitrogen fixation and ACC deaminase production to plant growth phenotypes, due to the complexities of the rhizosphere ecology (Saharan and Nehra 2011). Our plant growth promotion phenotypes, which were assayed under controlled agarose plate conditions in addition to soil in order to minimise such complexity, we propose that IAA is a major contributing factor to the stimulation of root development on *D. caryophyllus*. Importantly, the Murashige Skoog growth media used comprised high concentrations of soluble phosphate (12.5 mM) and nitrogen source (20 mM). Since *nif* gene expression (coding inter alia for the nitrogenase enzyme) and GDH/Pqq enzyme activity are stringently regulated and their induction requires no, or extremely low, nitrogen source (Dixon and Kahn 2004) and phosphate in the environment (Goldstein and Liu 1987), respectively, it is highly unlikely that these attributes account for the plant phenotypic outcomes in our experiments. Further, we inspected the SGM 81 genome and found no candidate *acdS* gene, required in other PGPR for ACC deaminase activity, excluding this PGP trait as a likely factor for our observations. We provided further evidence of IAA production by *Klebsiella* SGM 81 to account for the promotion of root growth, given the very similar root growth patterns of *D. caryophyllus* supplemented with commercial IAA or when inoculated with *Klebsiella* SGM 81 and evidencing tryptophan dependent IAA production proximal to root hairs in situ.

High density inoculum (10^8^ CFU.mL^−1^) exhibited negative effect on plant root development whether grown in soil or on MS Agar. A number of reasons could account for this, for example: (i) higher inoculation might lead to more IAA production, which would induce ethylene production and thus inhibit root growth and development (Husen et al. 2009); (ii) high density inocula could produce plant growth harmful metabolites inhibiting root development as shown in *Convolvulus arvensis* L (Sarwar and Kremer 1995); or (iii) high microbial titres may elicit plant defence mechanisms such as microbial associated molecular patterns (MAMPS) with adverse plant growth effects (Doornbos et al. 2012). However, because the high density *Klebsiella* SGM 81 inoculum resulted in poorer health of *D. caryophyllus* that correlated with a rapid decline in the rhizospheric bacterial population but not in a decline of endophytic bacteria, we propose that whatever the cause of poor plant health, the decline of rhizophere bacterial population is mainly attributable to a lack of positive effects of the plant towards bacteria, rather than a plant defence mechanism that would also target endophytic bacteria. This rational would be congruent with a strong mutualistic relationship between *D. caryophyllus* and rhizopheric bacteria*,* and specifically *Klebsiella* SGM 81.

We showed the presence of an *ipdC* gene in *Klebsiella* SGM 81 and demonstrated the production of substantial amounts of IAA in batch cultures supplemented with tryptophan. *ipdC* has been isolated and characterized from *Azospirillum brasilense* by (Costacurta et al. 1994), *Pseudomonas putida* by (Patten and Glick 2002b) and others and is generally associated with tryptophan dependent IAA production, fully consistent with our findings that IAA production was strictly dependent on tryptophan as substrate in batch cultures. It is worth noting that we found *Klebsiella* SGM 81 to produce substantially higher IAA amounts (190 μg.mL^−1^) compared with another *Klebsiella* strain (22.7 mg.L^−1^) studied by Sachdev et al. (2009), provided with the same concentration of tryptophan (0.1%). Interestingly, the IAA yields of *Klebsiella* SGM 81 observed in batch cultures are comparable with those reported for *Klebsiella* pnb8 (869 μg.mL^−1^) after addition of plant extract instead of tryptophan (Jasim et al. 2013). While tryptophan is commonly found in plant root exudates (Kravchenko et al. 2004), we did not directly determine tryptophan concentrations in *D. caryophyllus* exudates, which cannot be readily estimated in our experimental set up. The impact of the externally provided auxin IAA on root development is diverse and the production of IAA in plants complicates our understanding of IAA, which controls important plant developmental processes, such as tissue differentiation, cell enlargement and division (Teale et al. 2006). Externally applied IAA – either via direct IAA application or indirectly via IAA producing microbes, can have both plant growth promoting and plant deleterious effects and various microbial metabolic pathways to produce IAA have been associated with positive and negative plant outcomes (Spaepen and Vanderleyden 2011). We found that IAA, either applied directly or via inoculation with *Klebsiella* SGM 81 to have a generally positive effect on root growth of *D. caryophyllus*, apart from when applying very high titres of *Klebsiella* SGM 81 that are unlikely to occur in soil. This interpretation is consistent with the notion that most beneficial bacteria produce IAA via the so called indole-3-pyruvate pathway, for which *ipdC* is indicative, and which is thought to also be the most widespread IAA biosynthesis pathway in bacteria. We also found that a healthy *D. caryophyllus* correlated with the proliferation of the soil microbial community and specifically *Klebsiella* SGM 81 in the absence of other bacteria (MS-agarose). Taken together, our results strongly suggest that *Klebsiella* SGM 81 and *D. caryophyllus* have evolved a strong mutualistic relationship where IAA production by the former is likely to be a key determinant in this relationship.

We also showed that *Klebsiella* SGM 81 is an endophytic root coloniser of the *D. caryophyllus* apoplast. In support of this, *K. pneumoniae* has been reported to colonise the interior of maize roots and stems, with bacteria seen on the epidermis layer and pericycle region of the root, followed by relatively lower number of bacteria in the apoplast. (Chelius and Triplett 2000). Another report demonstrated a *Klebsiella* strain was able to colonise pepper roots, with cells on root hairs and lateral roots compared to the rhizoplane (Marasco et al. 2012). Our finding that the vast majority of *Klebsiella* SGM 81 appear to colonise the *D. caryophyllus* root and root hairs may suggest that IAA production and the resulting promotion of root and root hair growth could act locally through *Klebsiella* SGM 81 colonising the rhizoplane. Interestingly, exudation of plant derived tryptophan has been localised mainly near secondary graminacious root (Vacheron et al. 2013), so that tryptophan dependent bacterial IAA production could represent a beneficial biochemical ‘outsourcing’.

Our study focuses on mutualism between *Klebsiella* SGM 81 and *D. caryophyllus* and effect of IAA produced by SGM 81 on root architecture of plant. The exogenous IAA available to the plant roots exhibits the growth promotion of roots. We however could not validate if inoculation with SGM 81 along with its IAA triggers endogenous auxin synthesis by *D. caryophyllus* and together accounts for the change in root architecture, which constitutes future research study. Furthermore, the study of response of plant to the bacterial IAA at molecular levels will be valuable to understand the auxin induced plant – microbial interactions.

## Electronic supplementary material

ESM 1 General features of the *Klebsiella* SGM 81 genome**.** The summary of raw data with assemblies, trimmed reads and taxonomic distributions. (DOCX 11 kb)

ESM 2 **Supplementary Fig. 1** Gel electrophoresis*.* Agarose gel electrophoresis for conformation of PCR amplified putative ipdC gene showing ~1.7 kb band and 1 kb DNA ladder*.* **Supplementary Fig. 2** Schematic representation of plant experimental set up. Flowchart shows the steps conducted for plant study. **Supplementary Fig. 3** Protein sequence of SGM 81 from UniProt BLAST tool. ipdC derived amino acid sequence of indole pyruvate decarboxylase of *Klebseilla* SGM 81 and sequence similarity with indole pyruvate decarboxylase from *Klebsiella sp.* NFIX56*,* using UniProt BLAST. **Supplementary Fig. 4** Bacterial growth with different tryptophan concentration. The comparative effect of tryptophan concentration on bacterial growth calculated as CFU.mL^−1^ at 0, 24, 48, and 72 h in presence of 0.05% tryptophan and 0.5% tryptophan and no tryptophan (control). Error bar represents standard deviation of three experimental replicates. **Supplementary Fig. 5** In situ Salkowski staining on plant roots treated with different *Klebsiella* SGM 81 and control plants. Visual localisation of IAA using Salkowski reagent on roots of *Dianthus caryophyllus* treated with: (a) 10^5^ CFU.mL^−1^, (b) 10^8^ CFU.mL^−1^. The development of pinkish red colour proximal to root indicates the presence of auxin. The plant roots treated with 10^8^ CFU.mL^−1^ are not developed properly. Image 5c,d shows the pink colour development in tryptophan supplemented media and no colour development in absence of tryptophan respectively, after 24 h of bacterial inoculation in absence of plant. **Supplementary Fig. 6** Microscopy of root tissue to localise gfp tagged *Klebsiella* SGM 81. Colonisation of Carnation roots by *gfp* tagged *Klebsiella* SGM 81. Confocal microscopy was performed using 1 cm long root section. Images showing bacteria within epidermal *D. caryophyllus* cells (a), the root apoplasm (b) and non-treated control root cells and rhizoplane (c). Scale bar: 10 μm. (DOCX 3263 kb)

## Abbreviations

*Gfp*: Green florescent protein
IAA: Indole acetic acid
*ipdC*: Indole-3- pyruvate decarboxylase
PCR: Polymerase chain reaction
PGPR: Plant growth promoting rhizobacteria

## References

[CR1] Ahemad M, Khan MS (2011). Effects of insecticides on plant-growth-promoting activities of phosphate solubilizing rhizobacterium Klebsiella sp. strain PS19. Pestic Biochem Physiol.

[CR2] Ali B, Sabri A, Ljung K, Hasnain S (2009). Auxin production by plant associated bacteria: impact on endogenous IAA content and growth of Triticum aestivum L. Lett Appl Microbiol.

[CR3] Bais HP, Walker TS, Schweizer HP, Vivanco JM (2002). Root specific elicitation and antimicrobial activity of rosmarinic acid in hairy root cultures of Ocimum basilicum. Plant Physiol Biochem.

[CR4] Bent A (2006) Arabidopsis thaliana floral dip transformation method. Agrobacterium Protoc:87–104 10.1385/1-59745-130-4:87 16988336

[CR5] Bhardwaj D, Ansari MW, Sahoo RK, Tuteja N (2014). Biofertilizers function as key player in sustainable agriculture by improving soil fertility, plant tolerance and crop productivity. Microb Cell Factories.

[CR6] Bhattacharyya P, Jha D (2012). Plant growth-promoting rhizobacteria (PGPR): emergence in agriculture. World J Microbiol Biotechnol.

[CR7] Camacho C, Coulouris G, Avagyan V (2009). BLAST+: architecture and applications. BMC Bioinf.

[CR8] Chandra S, Rawat D, Chandra D, Rastogi J (2016). Nativity, phytochemistry, ethnobotany and pharmacology of dianthus caryophyllus. Res J Med Plant.

[CR9] Chelius MK, Triplett EW (2000). Immunolocalization of dinitrogenase reductase produced by Klebsiella pneumoniae in Association with Zea mays L. Appl Environ Microbiol.

[CR10] Cole JR, Chai B, Farris RJ (2005). The ribosomal database project (RDP-II): sequences and tools for high-throughput rRNA analysis. Nucleic Acids Res.

[CR11] Cormack BP, Valdivia RH, Falkow S (1996). FACS-optimized mutants of the green fluorescent protein (GFP). Gene.

[CR12] Costacurta A, Keijers V, Vanderleyden J (1994). Molecular cloning and sequence analysis of an Azospirilium brasilense indole-3-pyruvate decarboxylase gene. Mol Gen Genet MGG.

[CR13] Criscuolo A, Gribaldo S (2010). BMGE (block mapping and gathering with entropy): a new software for selection of phylogenetic informative regions from multiple sequence alignments. BMC Evol Biol.

[CR14] Darling AE, Mau B, Perna NT (2010). ProgressiveMauve: multiple genome alignment with gene gain, loss and rearrangement. PloS One.

[CR15] Datsenko KA, Wanner BL (2000). One-step inactivation of chromosomal genes in Escherichia coli K-12 using PCR products. Proc Natl Acad Sci.

[CR16] de Chaumont F, Dallongeville S, Provoost T, et al (2013) Icy: a user-friendly environment for algorithm development and deployment. IEEE, pp 1–5

[CR17] de Souza R, Ambrosini A, Passaglia LM (2015). Plant growth-promoting bacteria as inoculants in agricultural soils. Genet Mol Biol.

[CR18] Dixon R, Kahn D (2004). Genetic regulation of biological nitrogen fixation. Nat Rev Microbiol.

[CR19] Dobbelaere S, Croonenborghs A, Thys A (1999). Phytostimulatory effect of Azospirillum brasilense wild type and mutant strains altered in IAA production on wheat. Plant Soil.

[CR20] Doornbos RF, van Loon LC, Bakker PA (2012). Impact of root exudates and plant defense signaling on bacterial communities in the rhizosphere. A review. Agron Sustain Dev.

[CR21] Edgar RC (2004). MUSCLE: multiple sequence alignment with high accuracy and high throughput. Nucleic Acids Res.

[CR22] El-Khawas H, Adachi K (1999). Identification and quantification of auxins in culture media of Azospirillum and Klebsiella and their effect on rice roots. Biol Fertil Soils.

[CR23] Fournet-Fayard S, Joly B, Forestier C (1995). Transformation of wild type Klebsiella pneumoniae with plasmid DNA by electroporation. J Microbiol Methods.

[CR24] Goldstein A, Liu S (1987). Molecular cloning and regulation of a mineral phosphate solubilizing gene from Erwinia herbicola. Nat Biotechnol.

[CR25] Goswami D, Pithwa S, Dhandhukia P, Thakker JN (2014). Delineating *Kocuria turfanensis* 2M4 as a credible PGPR: a novel IAA-producing bacteria isolated from saline desert. J Plant Interact.

[CR26] Gravel V, Antoun H, Tweddell RJ (2007). Growth stimulation and fruit yield improvement of greenhouse tomato plants by inoculation with Pseudomonas putida or Trichoderma atroviride: possible role of indole acetic acid (IAA). Soil Biol Biochem.

[CR27] Grossman JD, Rice KJ (2012). Evolution of root plasticity responses to variation in soil nutrient distribution and concentration: Barley root plasticity. Evol Appl.

[CR28] Gyaneshwar P, James EK, Mathan N (2001). Endophytic colonization of rice by a diazotrophic strain of Serratia marcescens. J Bacteriol.

[CR29] Harikrishnan H, Shanmugaiah V, Balasubramanian N (2014). Optimization for production of Indole acetic acid (IAA) by plant growth promoting Streptomyces sp VSMGT1014 isolated from rice rhizosphere. Int J Curr Microbiol Appl Sci.

[CR30] Hayat R, Ali S, Amara U (2010). Soil beneficial bacteria and their role in plant growth promotion: a review. Ann Microbiol.

[CR31] Holt KE, Wertheim H, Zadoks RN (2015). Genomic analysis of diversity, population structure, virulence, and antimicrobial resistance in Klebsiella pneumoniae, an urgent threat to public health. Proc Natl Acad Sci.

[CR32] Husen E, Wahyudi AT, Suwanto A (2009). Soybean seedling root growth promotion by 1-aminocyclopropane-1-carboxylate deaminase-producing pseudomonads. Indones J Agric Sci.

[CR33] Jasim B, Jimtha CJ, Jyothis M, Radhakrishnan E (2013). Plant growth promoting potential of endophytic bacteria isolated from Piper nigrum. Plant Growth Regul.

[CR34] Jha CK, Saraf M (2011). In vitro evaluation of indigenous plant growth promoting rhizobacteria isolated from Jatropha curcas rhizosphere. Int J Genet Eng Biotechnol.

[CR35] Jha CK, Patel D, Rajendran N, Saraf M (2010). Combinatorial assessment on dominance and informative diversity of PGPR from rhizosphere of Jatropha curcas L. J Basic Microbiol.

[CR36] Jha CK, Annapurna K, Saraf M (2012). Isolation of Rhizobacteria from Jatropha curcas and characterization of produced ACC deaminase. J Basic Microbiol.

[CR37] Karnwal A (2009) Production of indole acetic acid by fluorescent Pseudomonas in the presence of L-tryptophan and rice root exudates. J Plant Pathol:61–63

[CR38] Kovach M, Phillips R, Elzer P (1994). pBBR1MCS: a broad-host-range cloning vector. BioTechniques.

[CR39] Kravchenko L, Azarova T, Makarova N, Tikhonovich I (2004). The effect of tryptophan present in plant root exudates on the phytostimulating activity of rhizobacteria. Microbiology.

[CR40] Mahl M, Wilson P, Fife M, Ewing W (1965). Nitrogen fixation by members of the tribe Klebsielleae. J Bacteriol.

[CR41] Marasco R, Rolli E, Ettoumi B (2012). A drought resistance-promoting microbiome is selected by root system under desert farming. PLoS One.

[CR42] Mastretta C, Taghavi S, Van Der Lelie D (2009). Endophytic bacteria from seeds of Nicotiana tabacum can reduce cadmium phytotoxicity. Int J Phytoremediation.

[CR43] Mayak S, Tirosh T, Glick B (1999). Effect of wild-type and mutant plant growth-promoting rhizobacteria on the rooting of mung bean cuttings. J Plant Growth Regul.

[CR44] Meuwly P, Pilet P-E (1991). Local treatment with indole-3-acetic acid induces differential growth responses in Zea mays L. roots. Planta.

[CR45] Patten CL, Glick BR (1996). Bacterial biosynthesis of indole-3-acetic acid. Can J Microbiol.

[CR46] Patten CL, Glick BR (2002). Role of pseudomonas putida indoleacetic acid in development of the host plant root system. Appl Environ Microbiol.

[CR47] Patten CL, Glick BR (2002). Regulation of indoleacetic acid production in pseudomonas putida GR12-2 by tryptophan and the stationary-phase sigma factor RpoS. Can J Microbiol.

[CR48] Peck SC, Kende H (1995). Sequential induction of the ethylene biosynthetic enzymes by indole-3-acetic acid in etiolated peas. Plant Mol Biol.

[CR49] Persello-Cartieaux F, Nussaume L, Robaglia C (2003). Tales from the underground: molecular. Plant Cell Environ.

[CR50] Riggs P, Moritz R, Chelius M et al (2002) Isolation and characterization of diazotrophic endophytes from grasses and their effects on plant growth. Nitrogen Fixat Glob Perspect N Y NY CAB Int:263–267

[CR51] Rueda-Puente E, Castellanos T, Troyo-Diéguez E (2003). Effects of a nitrogen-fixing indigenous bacterium (Klebsiella pneumoniae) on the growth and development of the halophyte salicornia bigelovii as a new crop for saline environments. J Agron Crop Sci.

[CR52] Sachdev DP, Chaudhari HG, Kasture VM, et al (2009) Isolation and characterization of indole acetic acid (IAA) producing Klebsiella pneumoniae strains from rhizosphere of wheat (Triticum aestivum) and their effect on plant growth 20329704

[CR53] Saharan B, Nehra V (2011). Plant growth promoting rhizobacteria: a critical review. Life Sci Med Res.

[CR54] Sarwar M, Kremer RJ (1995). Enhanced suppression of plant growth through production of L-tryptophan-derived compounds by deleterious rhizobacteria. Plant Soil.

[CR55] Seemann T (2014) Prokka: rapid prokaryotic genome annotation. Bioinformatics btu153

[CR56] Shanan NT, Higazy AM (2009). Integrated biofertilization management and cyanobacteria application to improve growth and flower quality of Matthiola incana. Res J Agric Biol Sci.

[CR57] Shiragur M, Shirol A, Reddy B, Kulkarni B (2004). Performance of standard carnation (Dianthus caryophyllus L.) cultivars under protected cultivation for vegetative characters. J Ornam Hortic.

[CR58] Singh RP, Jha P, Jha PN (2015). The plant-growth-promoting bacterium Klebsiella sp. SBP-8 confers induced systemic tolerance in wheat (Triticum aestivum) under salt stress. J Plant Physiol.

[CR59] Spaepen S, Vanderleyden J (2011). Auxin and plant-microbe interactions. Cold Spring Harb Perspect Biol.

[CR60] Stamatakis A (2014). RAxML version 8: a tool for phylogenetic analysis and post-analysis of large phylogenies. Bioinformatics.

[CR61] Teale WD, Paponov IA, Palme K (2006). Auxin in action: signalling, transport and the control of plant growth and development. Nat Rev Mol Cell Biol.

[CR62] Timmusk S, Grantcharova N, Wagner EGH (2005). Paenibacillus polymyxa Invades Plant Roots and Forms Biofilms. Appl Environ Microbiol.

[CR63] Tsavkelova EA, Cherdyntseva TA, Botina SG, Netrusov AI (2007). Bacteria associated with orchid roots and microbial production of auxin. Microbiol Res.

[CR64] Vacheron J, Desbrosses G, Bouffaud M-L (2013). Plant growth-promoting rhizobacteria and root system functioning. Front Plant Sci.

[CR65] Walker BJ, Abeel T, Shea T (2014). Pilon: an integrated tool for comprehensive microbial variant detection and genome assembly improvement. PLoS One.

[CR66] Weller DM, Raaijmakers JM, Gardener BBM, Thomashow LS (2002). Microbial populations responsible for specific soil suppressiveness to plant pathogens 1. Annu Rev Phytopathol.

[CR67] Wu Z, Peng Y, Guo L, Li C (2014). Root colonization of encapsulated Klebsiella oxytoca Rs-5 on cotton plants and its promoting growth performance under salinity stress. Eur J Soil Biol.

[CR68] Zulfitri A (2012) Plant growth promotion by IAA-producing rhizobacteria in ornamental plant propagation

