# Supplementary material for: Mutualism between Klebsiella SGM 81 and Dianthus caryophyllus in modulating root plasticity and rhizospheric bacterial density
Source: Plant Soil. 2017 Nov 8;424(1):273–88. doi: 10.1007/s11104-017-3440-5 (PMC6560813; doi:10.1007/s11104-017-3440-5)
Supplement: Supplementary file 1 — General features of the Klebsiella SGM 81 genome. The summary of raw data with assemblies, trimmed reads and taxonomic distributions. (DOCX 11 kb) [file 11104_2017_3440_MOESM1_ESM.docx]

| **Feature** | **Chromosome** |
| --- | --- |
| Size (bp) | 5336485 |
| G + C content (%) | 57.87 |
| Contigs | 62 |
| Length of longest contig (bp) | 2943174 |
| Most frequent family | Enterobacteriaceae |
| Most frequent genus (%) | 84.12 |

**Supplementary table**
