# Supplementary material for: Mutualism between Klebsiella SGM 81 and Dianthus caryophyllus in modulating root plasticity and rhizospheric bacterial density
Source: Plant Soil. 2017 Nov 8;424(1):273–88. doi: 10.1007/s11104-017-3440-5 (PMC6560813; doi:10.1007/s11104-017-3440-5)
Supplement: Supplementary file 2 — Supplementary Fig. 1 Gel electrophoresis. Agarose gel electrophoresis for conformation of PCR amplified putative ipdC gene showing ~1.7 kb band and 1 kb DNA ladder. Supplementary Fig. 2 Schematic representation of plant experimental set up. Flowchart shows the steps conducted for plant study. Supplementary Fig. 3 Protein sequence of SGM 81 from UniProt BLAST tool. ipdC derived amino acid sequence of indole pyruvate decarboxylase of Klebseilla SGM 81 and sequence similarity with indole pyruvate decarboxylase from Klebsiella sp. NFIX56, using UniProt BLAST. Supplementary Fig. 4 Bacterial growth with different tryptophan concentration. The comparative effect of tryptophan concentration on bacterial growth calculated as CFU.mL−1 at 0, 24, 48, and 72 h in presence of 0.05% tryptophan and 0.5% tryptophan and no tryptophan (control). Error bar represents standard deviation of three experimental replicates. Supplementary Fig. 5 In situ Salkowski staining on plant roots treated with different Klebsiella SGM 81 and control plants. Visual localisation of IAA using Salkowski reagent on roots of Dianthus caryophyllus treated with: (a) 105 CFU.mL−1, (b) 108 CFU.mL−1. The development of pinkish red colour proximal to root indicates the presence of auxin. The plant roots treated with 108 CFU.mL−1 are not developed properly. Image 5c,d shows the pink colour development in tryptophan supplemented media and no colour development in absence of tryptophan respectively, after 24 h of bacterial inoculation in absence of plant. Supplementary Fig. 6 Microscopy of root tissue to localise gfp tagged Klebsiella SGM 81. Colonisation of Carnation roots by gfp tagged Klebsiella SGM 81. Confocal microscopy was performed using 1 cm long root section. Images showing bacteria within epidermal D. caryophyllus cells (a), the root apoplasm (b) and non-treated control root cells and rhizoplane (c). Scale bar: 10 μm. (DOCX 3263 kb) [file 11104_2017_3440_MOESM2_ESM.docx]

**Supplementary Fig. 1**

**

**

***ipdC***

**1 kb**

**Supplementary Fig. 2**


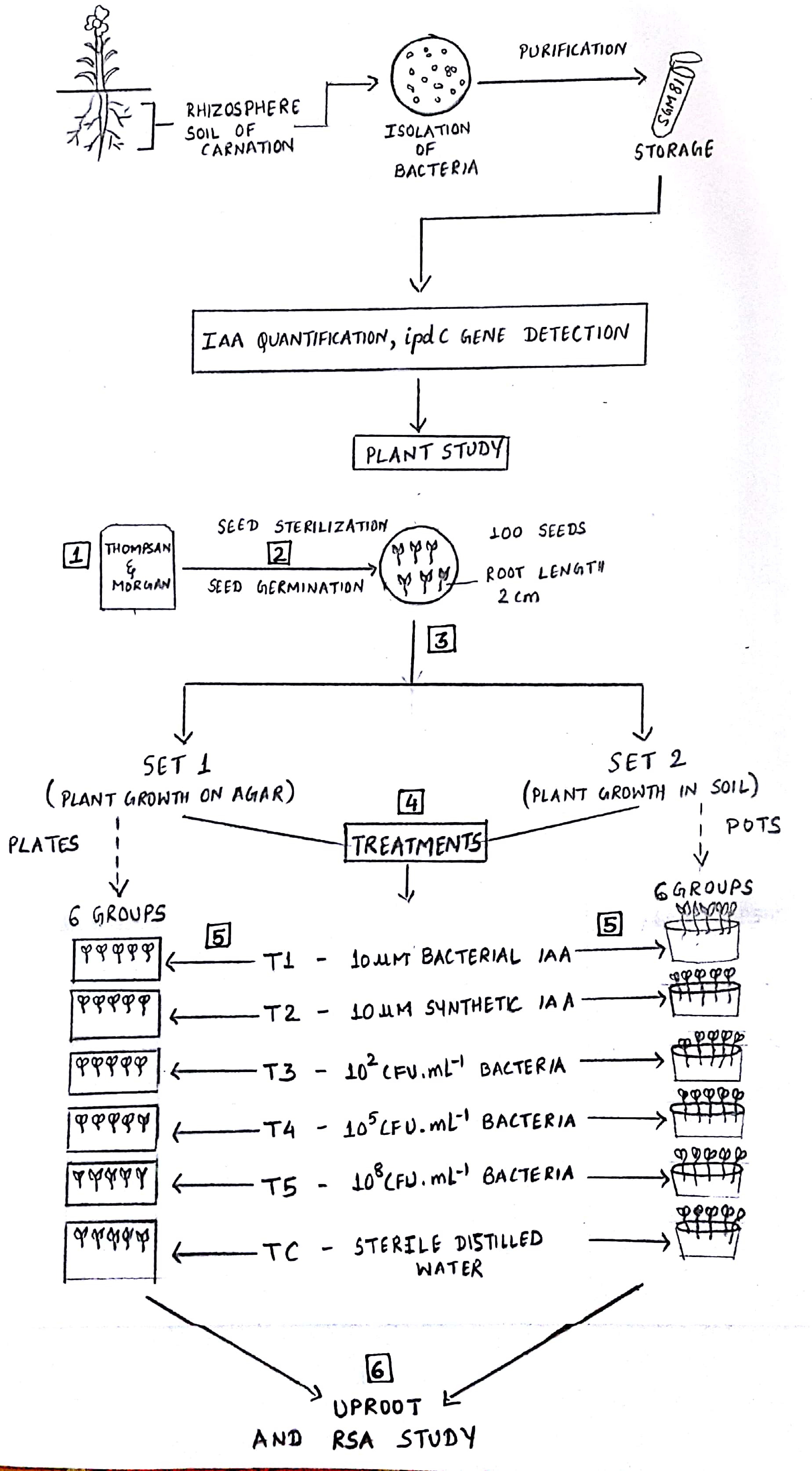


**Supplementary Fig. 3**

**Protein sequence of SGM 81 from UniProt BLAST tool**

MQPTYTIGDYLLDRLVDCGIDRLFGVPGDYNLQFLDRVIAHNALGWVGCANELNAAYAADGYARIKGAGALLTTYGVGELSALNGVAGSYAEHIPVLHIVGAPSTGAQQRGELLHHTLGDGDFRHFARMSEQITCSQALLTAGNACHEIDRVLRDMLTHHRPGYLMLPADVARAAAIAPAQRLLVEAAPADENQFAGFCEHASRLLRGSRRISLLADFLAQRYGLQNTLREWVAKTPVAHATMLMGKGLFDEQQRGFVGTYSGIASAPQTREAIENADTIICIGTRFTDTITAGFTQHLARDKTIEIQPFAVRVGDHWFSGVPMDQALAALMTLSAPLAAEWAAPQVVAPEVEEGADGELTQKNFWATVQGALRPGDIILADQGTAAFGIAALKLPSEASLIVQPLWGSIGFTLPAAYGAQTAAAERRVVLIVGDGAAQLTIQEMGSMLRDKQKPLILLLNNEGYTVERAIHGPEQRYNDIALWDWRRLPEAFAPDVASRCWRVTHTDELREAMAESITSDMLTLVEVMLPKMDIPDFLRAVTQALEERNSRV*

**
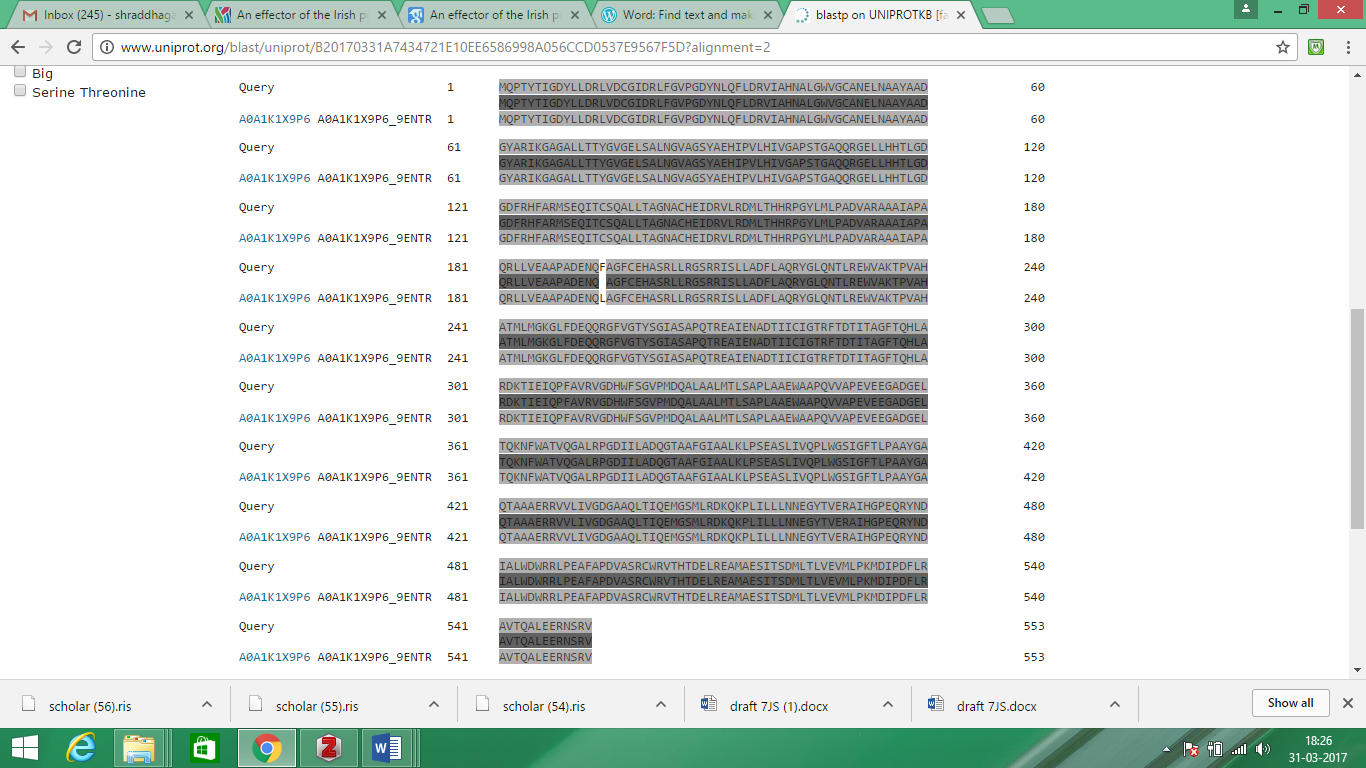
Sequence similarity of ipdC protein oh Klebseilla SGM 81 with Klebsiella sp NFIX56**

**Supplementary figure 4**

**Supplementary Fig 5**


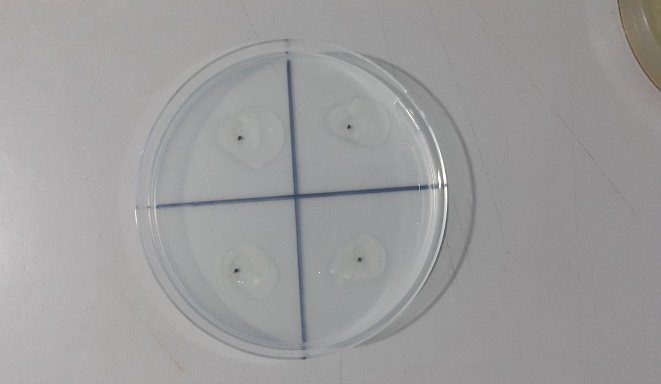

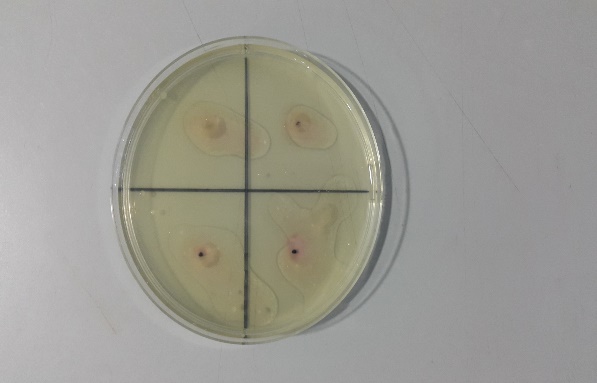

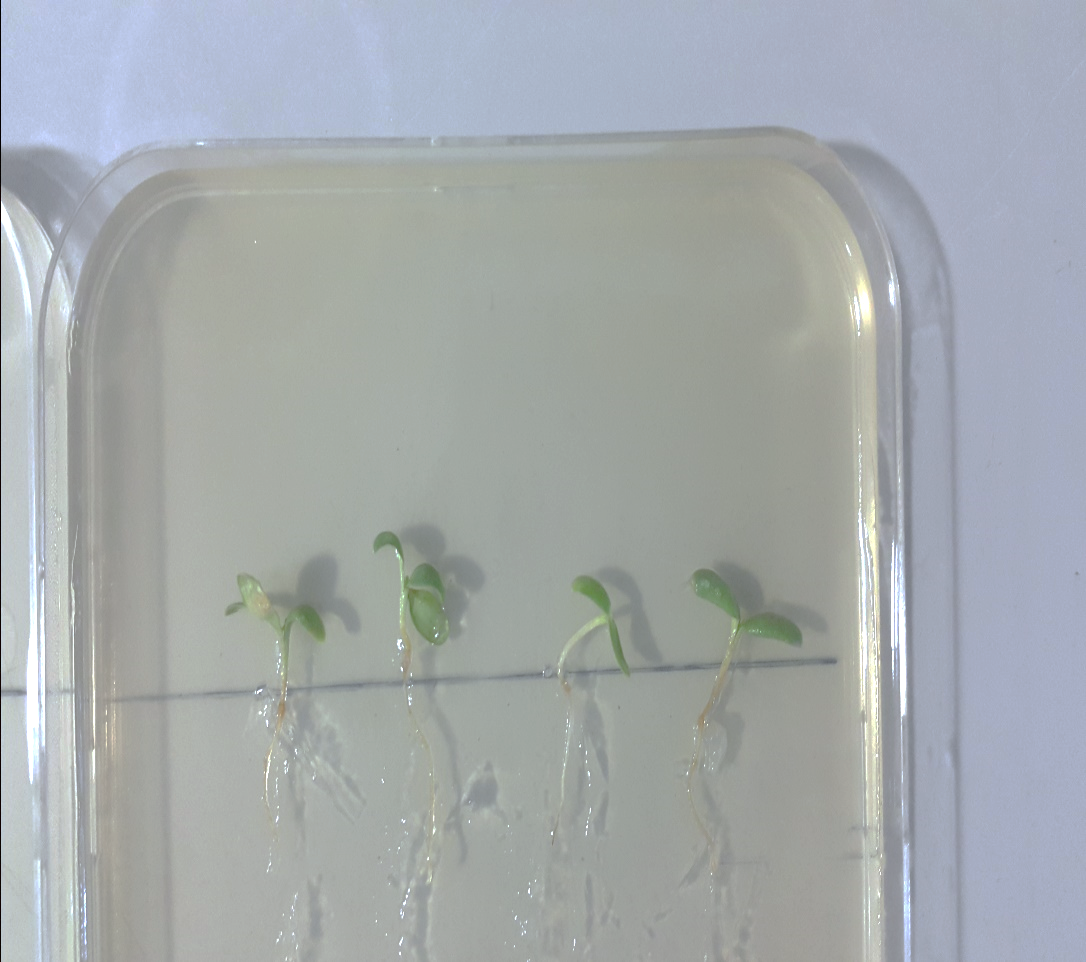

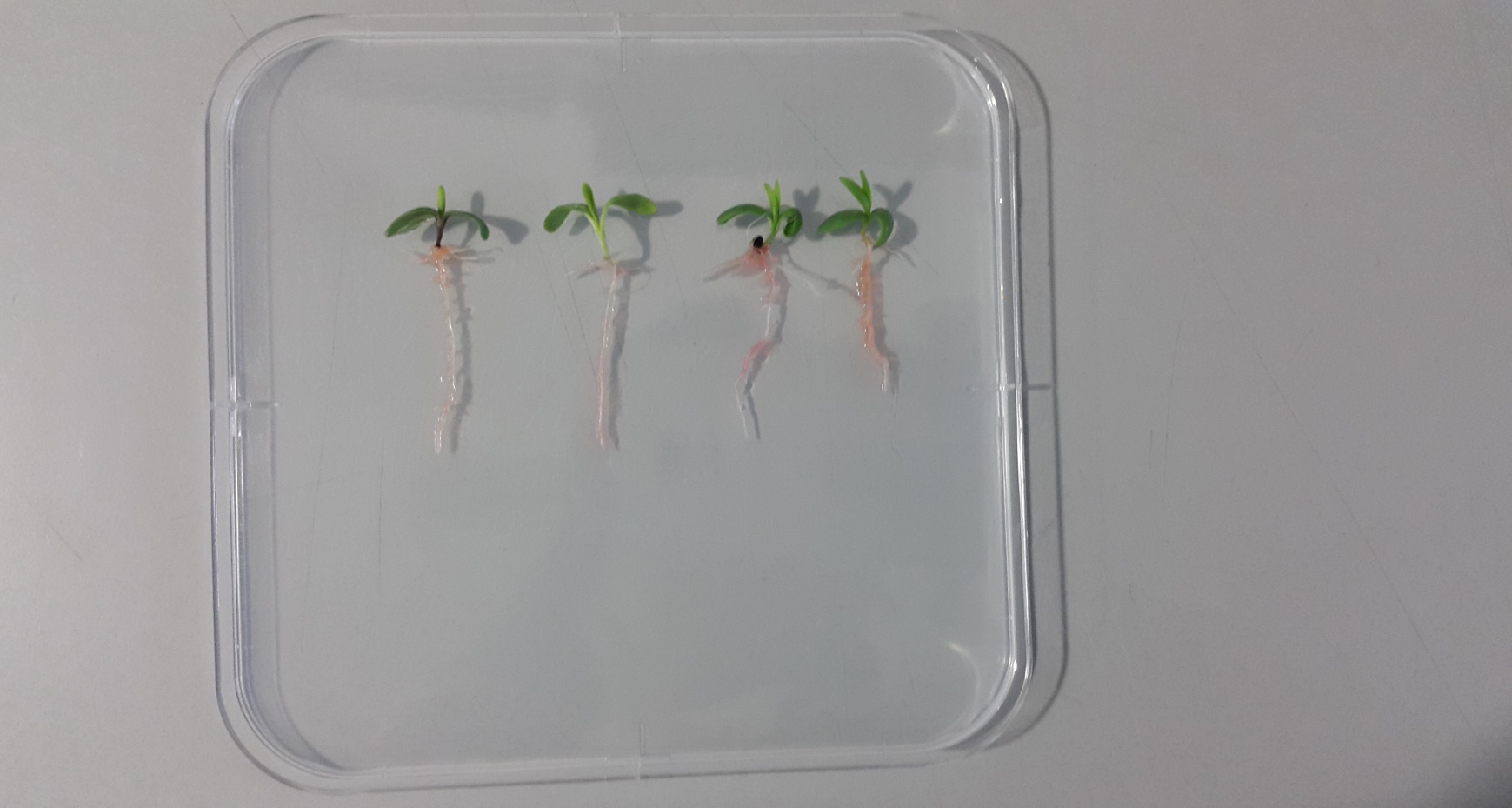


**(c)**

**(a)**

**(d)**

**(b)**

**(a)**

**Supplementary figure 6**


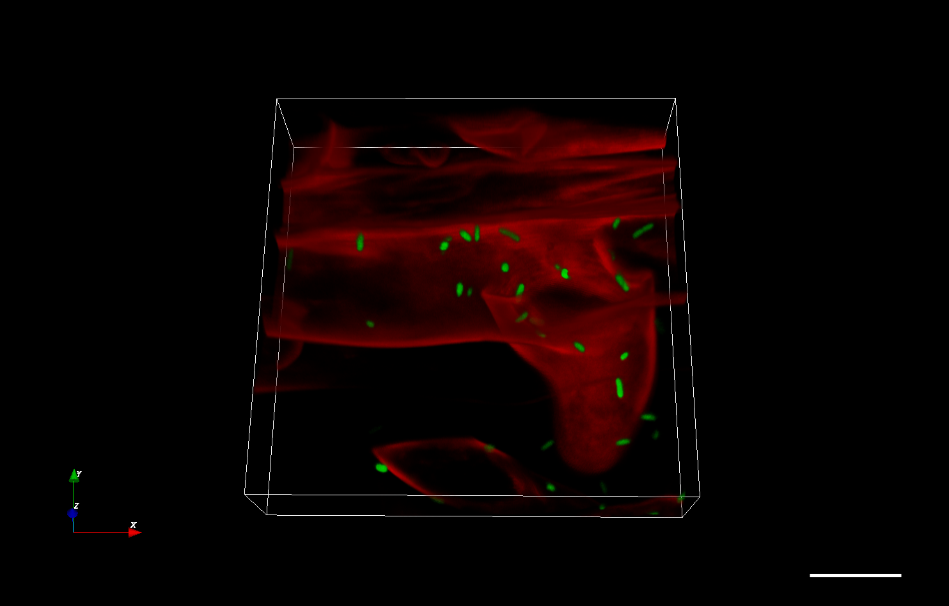


**(a)**

**(b)**


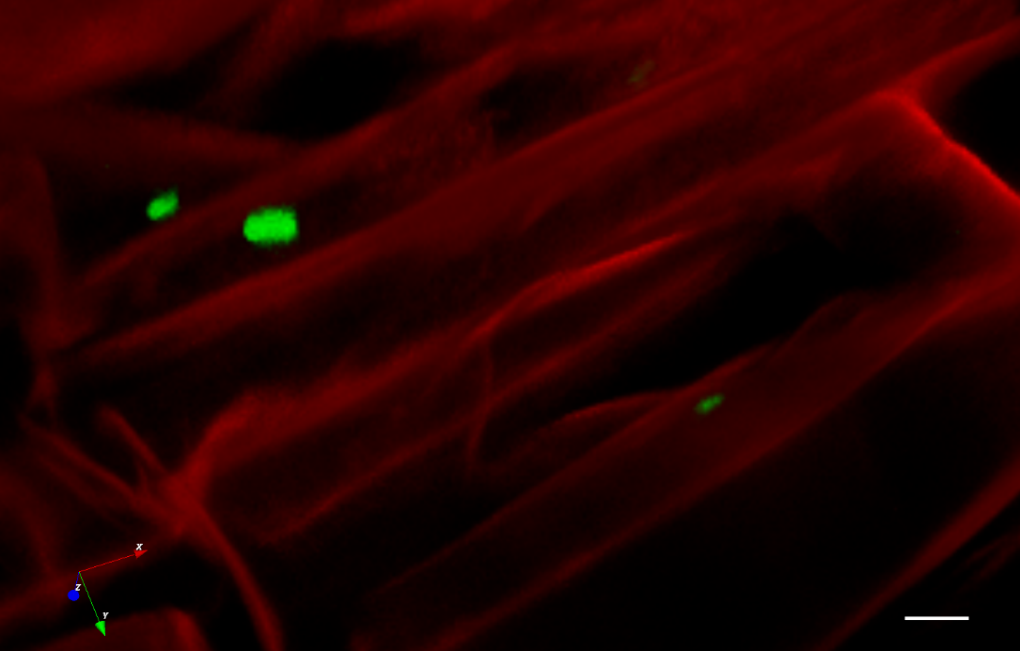


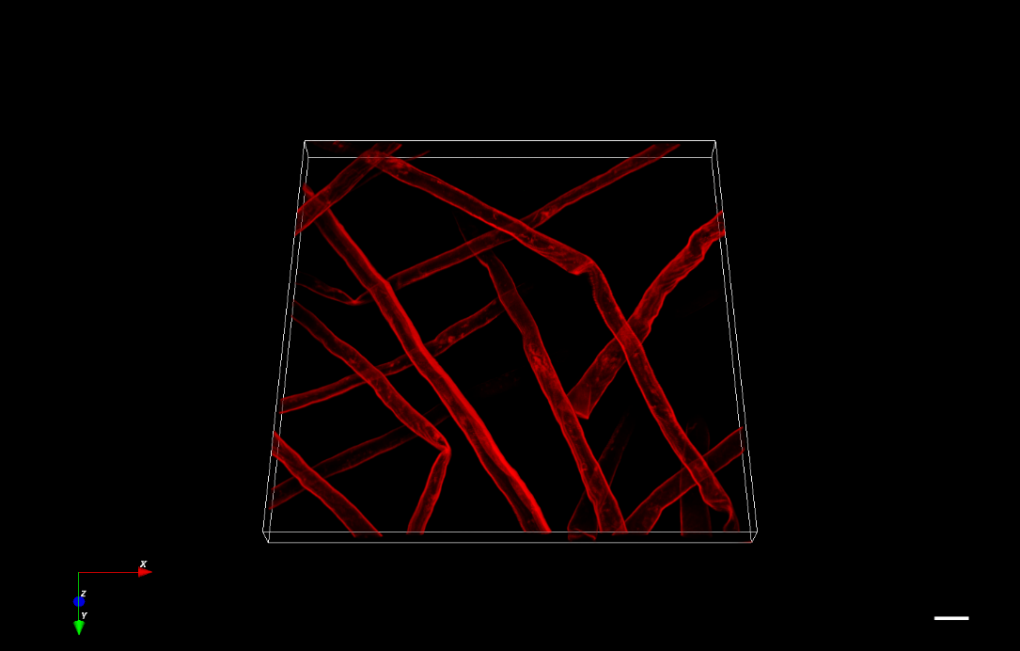


**(c)**

**(c)**
